# Incorporation of Cross-Linked Gelatin Microparticles To Enhance Cell Attachment and Chondrogenesis in Carboxylated Agarose Bioinks for Cartilage Engineering

**DOI:** 10.1021/acsami.5c00077

**Published:** 2025-04-07

**Authors:** Yi Qian, Yawei Gu, Fabian Tribukait-Riemenschneider, Ivan Martin, V. Prasad Shastri

**Affiliations:** † Institute for Macromolecular Chemistry, University of Freiburg, Freiburg 79104, Germany; ‡ Department of Biomedicine, Tissue Engineering Laboratory, University Hospital Basel, University of Basel, Basel 4031, Switzerland; § BIOSS—Centre for Biological Signalling Studies, University of Freiburg, Freiburg 79104, Germany

**Keywords:** gelatin microparticles, carboxylated agarose, bioink, cartilage engineering, cellular niches, 3D bioprinting

## Abstract

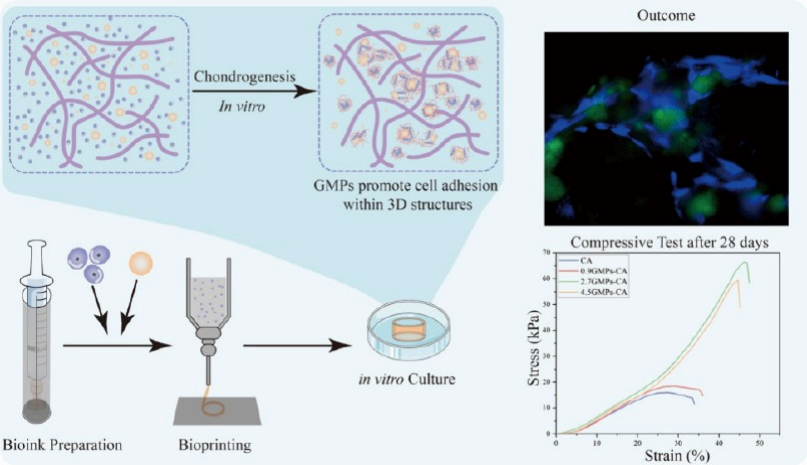

Due to the limited regenerative capacity of injured cartilage, surgical intervention using engineered cellular constructs or autologous cell implantation is the best accredited approach to prevent further degeneration and promote a regenerative microenvironment. Advancements in additive manufacturing present opportunities for graft customization through enhanced scaffold design. In bioprinting, an additive manufacturing process, the “bioink” serves as the medium to carry cells but also as a scaffold by imparting form and mechanical attributes to the printed object. In this study, the impact of cross-linked gelatin microparticles (GMPs) on rheological properties and printability of carboxylated agarose (CA) bioink as well as matrix deposition by human nasal chondrocytes (hNCs) was investigated. The introduction of GMPs yielded stiffer bioink formulations, with lower sol–gel transitions that retained the exceptional printability of CA. GMPs served as foci for the attachment of hNCs, improving cellular distribution and bridging the deposited extracellular matrix. After 4 weeks in chondrogenic culture, GMPs containing printed constructs showed enhanced toughness approaching that of the lower end of the spectrum of native cartilage tissue. The incorporation of proteinaceous microparticles might serve as a general concept to promote cellular function in polysaccharide-based bioinks and opens another avenue for engineering 3D-bioprinted microenvironments.

## Introduction

The increasing incidence of cartilage injury can be attributed to two factors: a more physically active lifestyle contributing to injury among younger individuals and an increasingly aging population.^1−3^ Cartilage injuries can stem from localized trauma, resulting in loss of cartilage integrity and lesions that can progress into osteoarthritis.^4^ Due to the lack of vasculature, cartilage tissue has poor regenerative capacity and surgical intervention is necessary, and can range from simple techniques such as microfracture^5^ to more complex treatments like transplantation of osteochondral plugs^6^ and cell therapy.^7^ Over the past two decades, in vitro engineered cartilage has been extensively explored as it can potentially provide an optimal solution as the quality of implanted tissue can be controlled and assessed prior to transplantation.^8−10^ Significant advances have been made in cartilage repair using cell transplantation,^11^ and new strategies to stimulate de novo cartilage formation have been demonstrated.^12^ However, when it comes to large defects, optimizing construct attributes such as shape and thickness to complement the defect can be important.

In polymer processing, 3D printing (3DP), a form of additive manufacturing, has risen to prominence as it offers rapid prototyping and a high degree of customization with respect to object dimension and mechanical properties. 3D-bioprinting (3DBP), a variation of 3DP focused on the printing of soft material such as hydrogels with biologically active elements such as cells, has been extensively explored for generating tissue models and organoids and is very promising as a method to engineer cellular constructs for tissue engineering. Large articular cartilage defects could benefit from 3DBP as engineered grafts can be mapped and customized in terms of thickness and contours to the defect.^13,14^ Additionally, 3DBP can improve outcomes through innovation in the design of scaffold matrix and customization of patient-specific cellular microenvironment.^15,16^

Two components are essential for bioprinting. A bioink capable of supporting cell attachment and matrix deposition with adequate mechanical properties for in vitro culture and implantation, and a reliable and readily accessible source of cells. In this regard, three main sources of cells have been explored for cartilage engineering, which are human mesenchymal stem cells derived from bone marrow (or adipose tissue),^17,18^ articular chondrocytes,^16^ and more recently, human nasal-septal derived chondrocytes (hNCs).^19^ Among them, hNCs have shown promising outcomes in human clinical trials.^19^ Interestingly, data from the clinical studies database point to a better outcome with chondrocytes than adipose or marrow-derived stem cells.^11^

To date, considerable effort has been directed toward bioprinting cartilage constructs from human cells but with only moderate success. As hydrogels are the most common materials used for bioprinting, one of the major concerns in engineering cartilage is the structural stability. To address this, composites of 3D printed thermoplastic such as polycaprolactone infused with hydrogel containing cells^20^ or bioink coprinted with polylactic acid^21^ have been explored. While such composite scaffolds can maintain their shape in vivo, the two-material construction can lead to variations in degradation and tissue inhomogeneity. A monomaterial construct derived from a single phase that can both support tissue formation and structural integrity can be advantageous.

**Figure 1:**
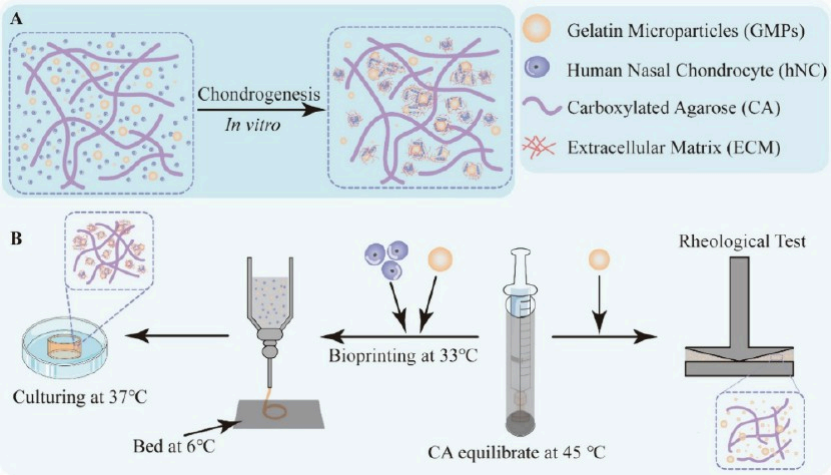
(A) Schematic illustration of the hypothesis: The bioink, formulated with carboxylated agarose and cross-linked gelatin microparticles, can enhance human nasal chondrocytes’ adhesion within a 3D environment, thus promoting the chondrogenesis process. (B) The workflow of the study.

Currently, several hydrogel forming materials are being explored as bioinks in microextrusion printing, including animal source-based bioactive polymers, such as gelatin,^22^ collagen,^13^ and their modified forms; or plant-source-based polymers, such as alginate,^21^ agarose, and their derivatives.^23^ Carboxylated agarose (CA), a chemically modified derivative of agarose, a red marine algae-derived polysaccharide, although biologically inert and lacking in cell attachment motifs, is quite promising as an extrudable carrier for chondrocytes, and possesses many essential attributes for printing cell-laden structures. These attributes include sol–gel transition that can be realized at or below physiological temperature, shear-thinning behavior over a wide range of concentrations, printability at low extrusion pressures, and capability in printing high concentrations of cells without appreciable loss in cell viability.^24−27^ A more recent effort in 3D printing of osseous tissue of predefined shape using a CA-based bioink has shown that CA-printed cartilaginous templates could maintain their structure for at least 12 weeks in vivo.^15^ Encouraged by these findings, in the present study we aimed to further extend the potential of CA for cartilage tissue engineering. Although CA-based bioinks could induce MSC aggregation inside the 3D constructs, initiating chondrogenic differentiation,^15,28^ the efficiency of chondrogenesis can be further improved through enhanced cell attachment. Toward this objective, we postulated that the introduction of a biopolymer bearing cell adhesion motifs could provide cell adhesion sites in the CA environment, improving outcomes. To explore this hypothesis, in this study, the impact of incorporation of gelatin microparticles (Figure 1A) on the rheological properties and printability of CA bioink, as well as its influence on hNC attachment and matrix production, was investigated (Figure 1B).

## Results and Discussion

### Gelatin-CA Blends

Gelatin is a denatured form of collagen that has been currently in use in several United States FDA-approved products, for example, Gelfoam and SurgiFoam, which are used as hemostats. Gelatin presents accessible RGD motifs^29^ that are otherwise cryptic in type-1 collagen.^30^ Therefore, we first explored a straightforward approach of blending gelatin with CA to determine if it can induce cell attachment. Since gelatin is also a thermoresponsive polymer and undergoes gelation below physiological temperatures, it is compatible with the sol–gel transitions in CA. Simply, blending CA with gelatin type A (GelA) in different w/w ratios yielded soft hydrogels that were, however, incapable of promoting the spreading of NIH-3T3 fibroblasts and yielded cell clusters over time (Figure S1). In contrast, CA-GelA hydrogels upon cross-linking with glutaraldehyde were very efficient at supporting fibroblast attachment and proliferation (Figure S2). Since unreacted glutaraldehyde is toxic to cells, cross-linking CA-GelA bioink postprinting in the presence of cells is not practical. Based on this observation, we posit that the incorporation of gelatin as cross-linked microparticles (GMPs) could provide attachment foci for cells and function as physical cell niches (Figure 1A).

### GMP Synthesis and Characterization

**Figure 2:**
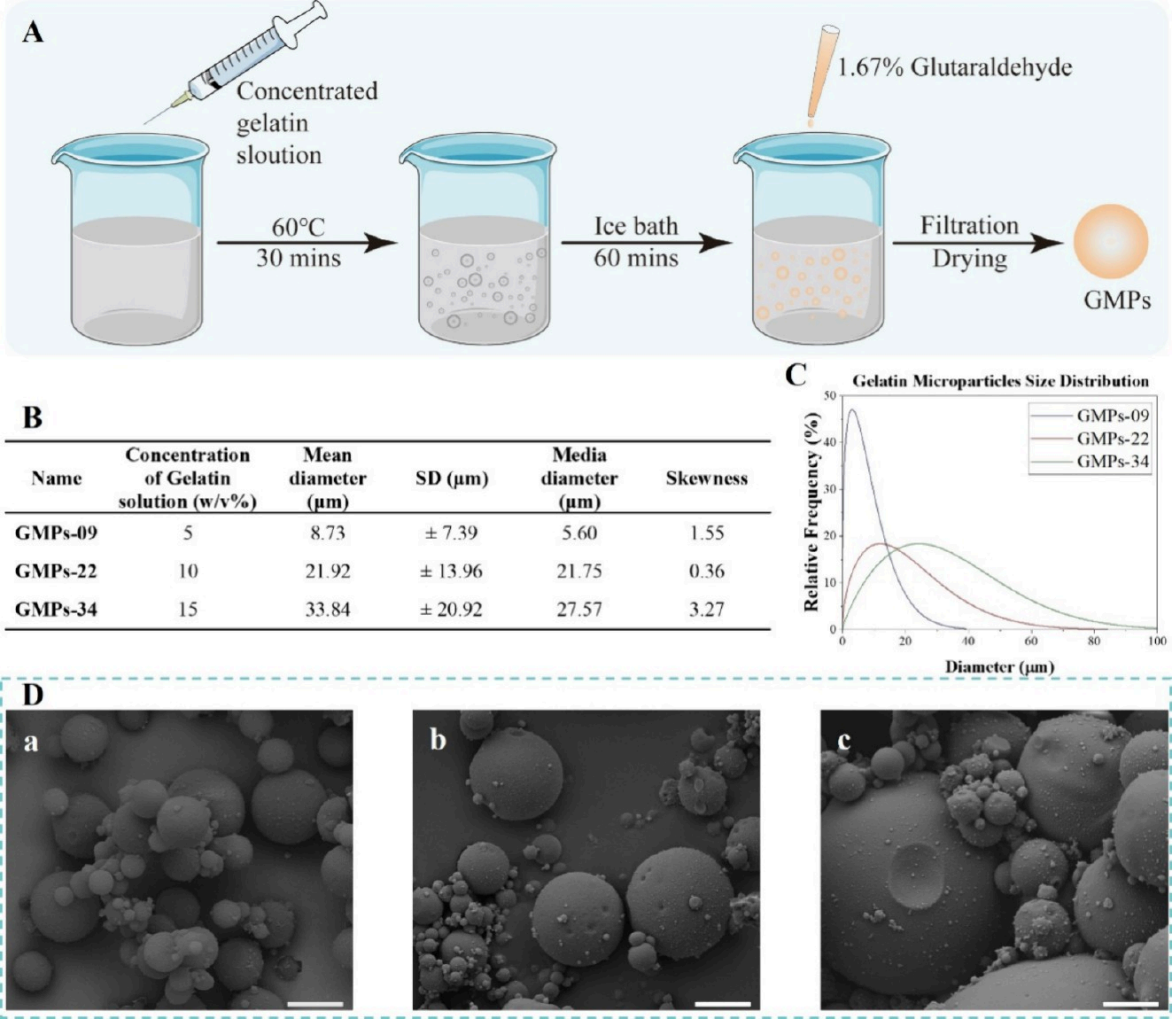
Synthesis and characterization of gelatin microparticles (GMPs). (A) Schematic illustration of GMPs synthesis. (B,C) Analysis of particle size and size distribution of GMPs synthesized with varying gelatin concentrations (*N* = 3). (D) Representative SEM images of GMPs prepared using gelatin concentration of (a) 5 w/v%, (b) 10 w/v% and (c) 15 w/v%. Scale bar = 15 μm.

Since gelatin is a highly water-soluble protein and has no solubility in oil, a common approach to produce GMPs dispersions is via the water-in-oil (w/o) emulsion method^31^ followed by cross-linking with glutaraldehyde (as illustrated in Figure 2A). By adjusting concentrations of the gelatin solution, GMPs with sizes ranging from 8.73 ± 7.39 to 33.84 ± 20.92 μm could be produced (Figure 2B). The GMPs are denoted based on their average size as GMPs-09, GMPs-22, and GMPs-34, respectively. The size distribution profile shown in Figure 2C indicates that GMPs-22 and GMPs-34 possess a broader distribution compared to GMPs-09. Factors such as concentration and type of emulsifier,^32^ mechanical agitation,^31^ and temperature^33^ can all contribute to a broad size distribution. In addition, the w/o process produced small particles with a diameter under a micrometer. Scanning electron microscopy (SEM) images revealed that these smaller GMPs associated strongly with larger GMPs decorating the surface of the larger particles in all groups (Figure 2D), possibly due to differences in net surface charge^34^ and/or factors similar to those at play in the formation of Pickering emulsions.^35^ In the future, one could minimize such artifacts using GMPs with near-unimodal size distribution. Another aspect that deserves consideration is the cytotoxicity of glutaraldehyde. It has been reported that at a concentration of 2.4 μg/mL, glutaraldehyde can reduce cellular viability by 90%.^36^ Nonetheless, it is important to point out that what matters is the concentration of the residual glutaraldehyde. The St. Jude’s Epic heart valve, which is approved for human use by the United States Food and Drug Administration and has shown satisfactory performance in human clinical trials, is in fact a glutaraldehyde cross-linked porcine heart valve.^37^ Moreover, glutaraldehyde cross-linked gelatin drug delivery devices have been successfully explored in investigating signaling pathways in human marrow-derived mesenchymal stem cells without any adverse outcomes on cell viability and function.^38,39^ In order to ensure minimal glutaraldehyde in the GMPs, GMPs were thoroughly rinsed until no residual glutaraldehyde was undetectable (Figure S3). Additionally, as demonstrated in Figure S2, this rinsing procedure enabled NIH 3T3 cells to adhere to the surface of cross-linked CA-GelA hydrogels and proliferate, indicating that the nondetectable residual glutaraldehyde level in the culture media was noncytotoxic.

An important aspect of using GMPs dispersion is the ability to ascertain the true mass fraction in the bioink solution, as the concentration of GMPs will influence the rheological and mechanical properties of bioinks. To achieve a more reliable quantification of the mass of GMPs in the bioink formulation, we carried out an experiment to correlate the actual mass of GMPs in a dispersion. Using a suspension of GMPs in DPBS at a known concentration (75 mg/mL), the required volume to obtain a specific mass of GMPs was determined (Table S1). By comparing the actual mass in aliquots to the theoretical value, an empirical relationship was established (Figure S4) that allowed us to account for the loss of GMPs due to precipitation and aggregation and compensate for this discrepancy during the bioink formulation.

### GMPs Do Not Impact the Printability of CA-Based Bioink

**Figure 3:**
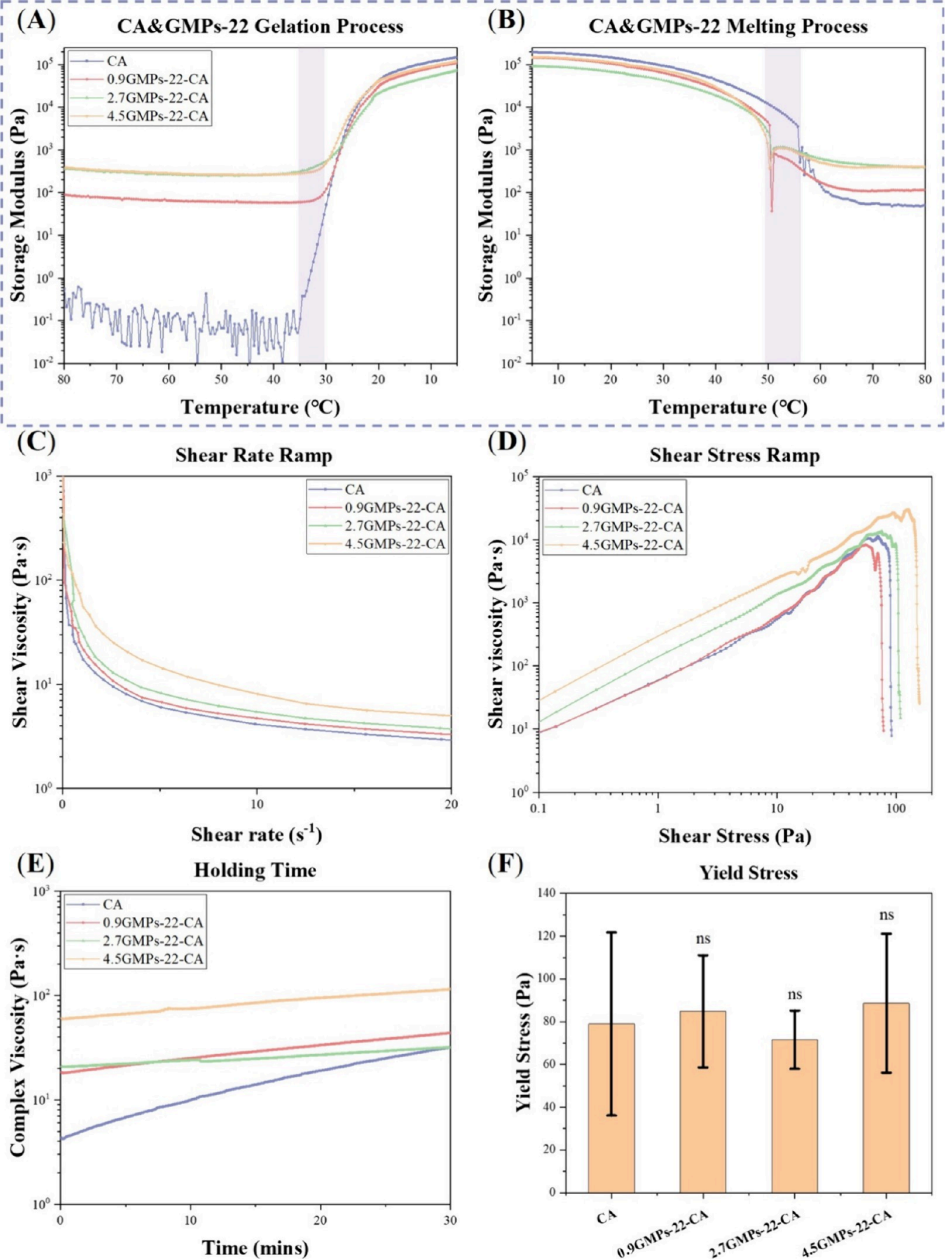
Effect of GMPs on rheological characteristics of CA bioink. Temperature sweep showing (A) sol–gel and (B) gel–sol transition of the bioinks. The fluctuations in the CA curve before sol–gel transition is due to the onset of dynamic physical cross-links. Shear viscosity of bioinks as a function of shear rate (C), and shear stress (D), complex viscosity as a function of holding time (E) at 33 °C (printing temperature), and (F) yield stress derived from data presented in (D) represented as mean ± standard deviation of the mean (*N* = 3, ns: no statistically significant difference). The concentration of CA in all bioinks was 80 mg/mL (8% w/v).

Since CA-based bioinks possess specific rheological characteristics for extrusion-based printing, namely, shear-thinning and predictable sol–gel transitions near physiological temperatures, it is essential to evaluate the impact of GMPs’ incorporation on these rheological characteristics. Building on our prior studies, CA with a *G*′ at 1067.5 Pa was selected as the foundation for the bioink formulation owing to its appropriate gelling profile and its ability to preserve the integrity of printed structures.^15,27^ A series of characterizations simulating the preparation and printing process were performed on GMPs-CA bioinks (0.9, 2.7, and 4.5 mean 0.9% w/w, 2.7% w/w, and 4.5% w/w of GMPs, respectively). In the cyclic temperature sweep test from 90 to 4 °C, all the bioink formulations underwent a sol–gel and a gel–sol transition, which could reveal the influence of GMPs on the viscoelastic behavior of GMPs-CA bioinks. Upon the introduction of GMPs, the *G*′ of all GMPs-CA bioinks in solution state (prior to onset of gelation) exhibited a two-order-of-magnitude increase compared to the CA-only bioink. Furthermore, the *G*′ of GMPs-CA bioinks increased with the mass fraction of GMPs, regardless of GMPs’ size (Figure 3A, Table S2, *G*′ at 45 °C). Another notable change was the reduction in sol–gel transition temperatures (*T*_sol–gel_), which dropped to a range of 29.6–33.4 °C from the original 35 °C of CA. As insoluble microparticles in CA solution, GMPs could potentially disrupt the entanglement of CA polymer chains during cooling, leading to a reduced storage modulus (*G′*) at lower temperatures and a quicker disentanglement upon heating (Figure 3B). Nonetheless, the gelling patterns of GMPs-CA bioinks showed similarity to that of CA, and the melting temperature exceeded the physiological temperature, thus ensuring the printability of GMPs-CA. GMPs with the other sizes (GMPs-09, GMPs-34) exhibited an analogous effect on the gelling and melting profiles (Figure S5).

In our printing protocol, bioinks containing GMPs-09 were observed to clog the nozzle easily irrespective of the volume fraction. To streamline our experiments and reduce the impact of variables, we selected GMPs with an average diameter of 22 μm, i.e., GMPs-22, for all further testing, and are henceforth referred to as GMPs. Additionally, through an optimization study, 33 °C was identified as the optimal printing temperature for GMPs-CA bioinks, which we adopted for the subsequent rheological assessments. We found that while the shear viscosity increased with the volume fraction of GMPs, GMPs-CA bioinks retained shear-thinning behavior similar to CA bioinks (Figure 3C). Consequently, the yielding stress of GMPs-CA bioinks demonstrated the same trend (Figure 3D,E). Bioink stability is another important aspect of printing. GMPs-CA bioinks showed only a mild rise in viscosity during a holding time of 30 min compared to the CA bioinks, which ensured persistent printability with subtle changes in extrusion pressure (Figure 3F).

### GMPs Are Dispersible within CA Hydrogels Bioinks

**Figure 4:**
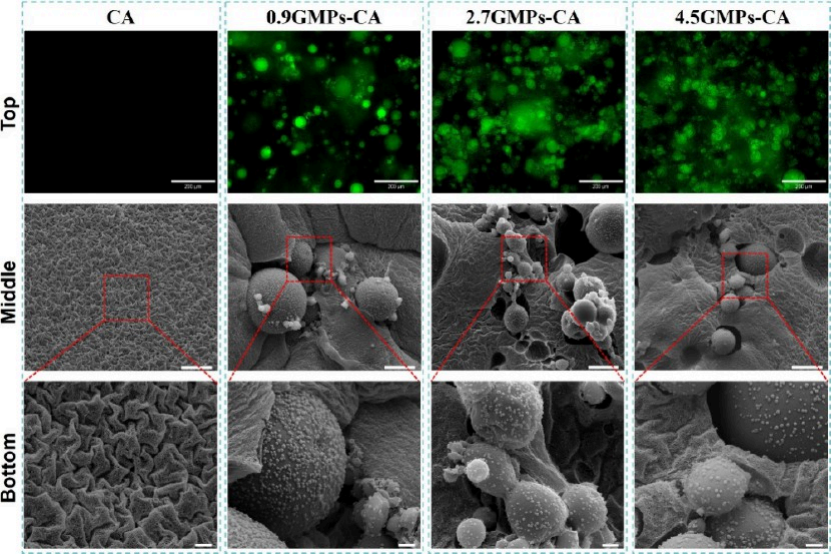
Distribution of GMPs within CA hydrogels. Top panel: Fluorescence microscopy images of the GMPs-CA hydrogel showing the distribution of GMPs which exhibit autofluorescence. Middle and Bottom: SEM images of critical-point dried CA and GMPs-CA hydrogel showing the GMPs’ remain intact and reside within the hydrogel. SEM images in the bottom panel are magnification of the area bounded by the red square in the middle row. Scale bar: 200 μm for top row, 15 μm for middle row and 2 μm for bottom row.

Since cross-linking of protein with glutaraldehyde occurs through Schiff base formation, it would cause autofluorescence over a large excitation wavelength range.^40^ This allowed us to observe the distribution of GMPs throughout the CA hydrogels by fluorescence microscopy. We found that GMPs were generally distributed uniformly throughout the hydrogel in spite of some agglomeration (Figure 4 Top). Furthermore, SEM images of the GMPs-CA hydrogels processed by critical point drying revealed that GMPs were embedded in the CA-formed architecture without breaking down their structural integrity even at high concentrations (Figure 4 Middle and Bottom). As observed in the dispersions, larger GMPs were decorated with smaller GMPs, which increased the surface roughness. This heightened roughness likely amplifies interparticle friction,^41^ as supported by rheological characterization showing elevated shear viscosity. The increased shear viscosity is advantageous for extrusion-based printing, as it promotes filament stability by resisting droplet formation. Furthermore, surface roughness has been shown to influence cell adhesion, cell morphology, F-actin assembly^42,43^ and protein adsorption.^44^ However, the optimal roughness range of GMPs for maximizing cell attachment and proliferation remains unclear and warrants further investigation.

### GMP-CA Bioinks Are Amenable to Printing Complex Structures

**Figure 5:**
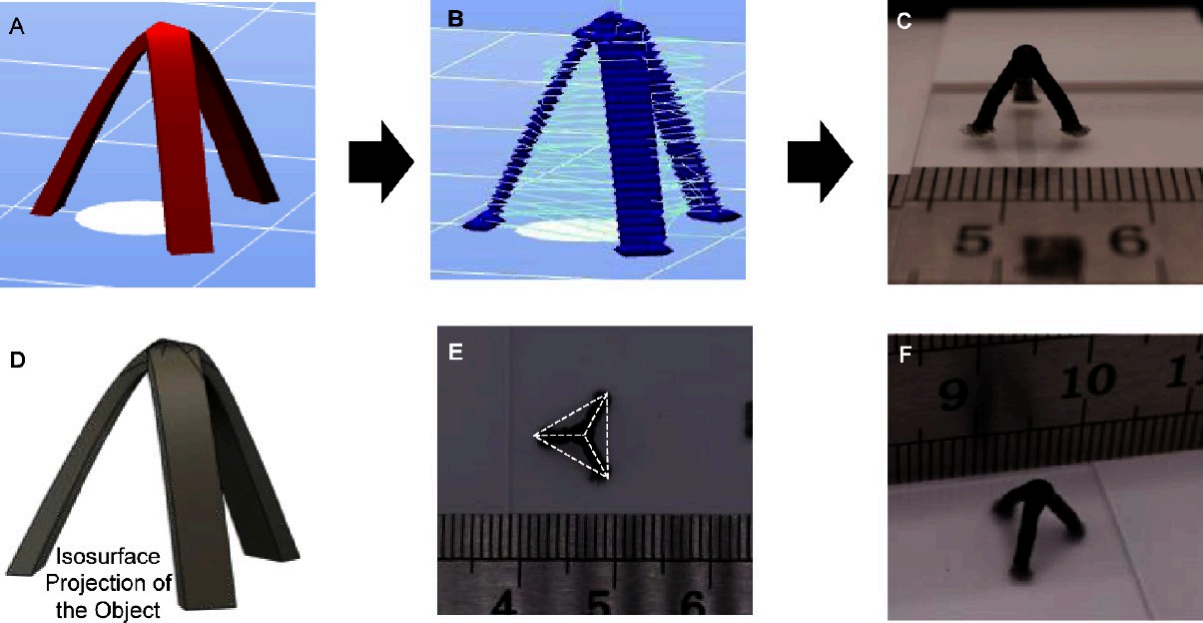
Trigonal pyramidal structure printed using 2.7GMPs-CA bioink. (A) CAD rendering of the structure, (B) print path (gcode) of the structure obtained after processing using Slic3r, (C) photograph of the object in the transverse plane, (D) isosurface projection of the object for comparison, (E) top view of the printed object superimposed with a trigonal pyramidal frame showing the accuracy of the print and the convergence of the three legs (struts) at the apex of the pyramid, and (F) oblique angle view of the structure illustrating the height of the printed object.

We have previously shown that CA bioinks are capable of yielding free-standing structures of complex geometries.^15,27^ In order to investigate if the introduction of GMPs into the CA preserves this unique attribute, the printing of a trigonal, pyramidal structure comprising three struts that converge into a point was undertaken (Figure 5A,B). This structure presents several challenges as it involves printing: (1) elements with high aspect ratios, (2) a structure that has overhangs and a moving center of gravity, and (3) a structure that requires convergence of three elements into a point at the center above the triangular base. In order to ensure white light photography of the structure, a minute amount (0.2% by weight in a total solid content of 8.7 wt %) of activated charcoal was added to the bioink. The presence of GMP did not hinder the printing of this complex structure (Figure 5C), and all the design objectives (Figure 5D) were met, as evident by the convergence of the three struts at a point ∼1 cm above the plane of the triangle (Figures 5E,F). The printing of this self-supported structure was accomplished in a very short printing time, suggesting that the bioink exhibits rapid gelation and adhesion. Additionally, the low thickness-to-height ratio of the structure is proof that the structure can bear its own weight. The ability to print thin legs demonstrated that during the dispensation of the bioink, the nozzle tip does not adhere to the print, thereby avoiding movement of the structure during printing. Furthermore, the single perimeter with no fill demonstrates the accuracy of the print and affirms the exceptional layer-to-layer adhesion in the GMP-CA bioink. In its totality, this example confirms the suitability of GMP-CA bioinks for printing complex and anatomically relevant structures.

### GMPs-CA Bioinks Support Printing of hNCs and Serve as Attachment Sites

To further investigate how GMPs affect cell behavior, hNCs, transduced to constitutively express blue fluorescence protein (BFP), were printed at a concentration of 3 × 10^7^ cells/mL using either CA bioink or GMPs-CA bioinks with varying GMPs ratios. The selected structure for printing was a square sheet measuring 5 mm in length on each side and 0.5 mm in thickness. BFP was chosen due to the weak signal overlap with the autofluorescence emitted by GMPs at the excitation and emission wavelengths specific to BFP.

**Figure 6:**
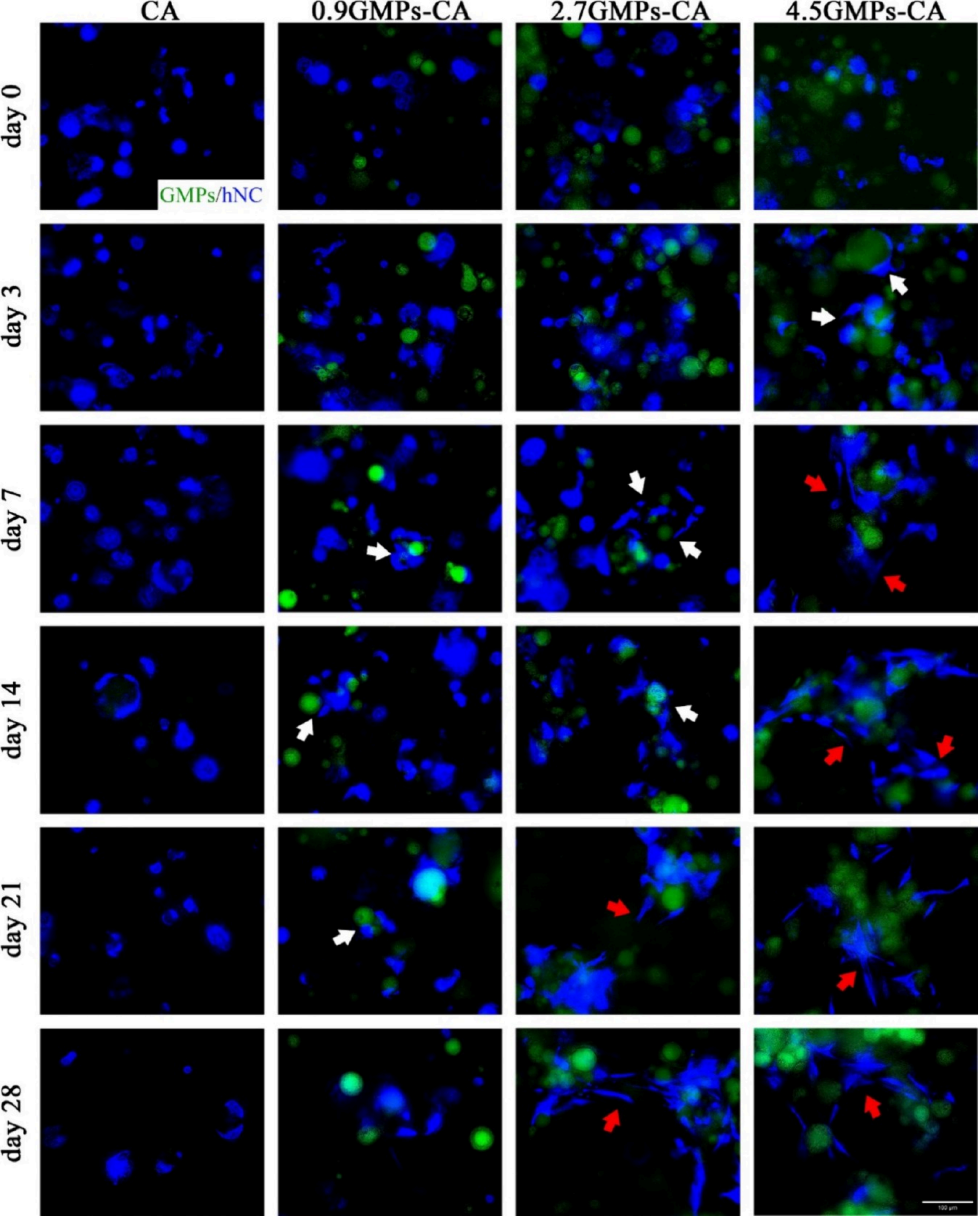
Interaction between GMPs and hNCs within the CA environment. BFP-expressing hNC (blue) (3 × 10^7^ cells/mL) were printed into square sheets (5 mm × 5 mm × 0.5 mm) and were cultured in chondrogenic differentiation medium up to 28 days. GMPs provided cell attachment foci (white arrows) and promoted formation of cell niches (red arrows) as evidenced by the cells bridging several GMPs. Scale bar = 100 μm.

It was observed that hNCs either aggregated into spherical clusters or remained as individual cells (small round shape) throughout the 28-day culture period in the printed CA constructs (Figure 6, left column). In contrast, within GMPs-CA printed constructs, hNCs surrounding the GMPs displayed morphology consistent with cell spreading. In 0.9GMPs-CA and 2.7GMPs-CA printed constructs, cell spreading was first observed on day 7, whereas in 4.5GMPs-CA constructs, it was detectable as early as 3 days postprinting (Figure 6, in the middle and right columns, indicated by the white arrows). With culturing, hNCs proliferated, merged, and formed “bridges” among GMPs. The more concentrated the GMPs, the more cell bridges occurred (Figure 6, in the middle and right columns, indicated by the red arrows). The establishment of cell bridges among GMPs validated our hypothesis that within the 3D hydrogel environment, GMPs functioned as focal sites for cell adhesion.

### GMPs Function as a Foci for ECM Deposition within the Bioinks

Building on the above encouraging results, we then evaluated the performance of GMPs-CA bioinks in the biofabrication of cartilaginous tissue in vitro. For these experiments, the concentration of hNCs was increased to 6 × 10^7^ cells/mL, and a ring-shaped structure (6 mm in outer diameter, 5 mm in inner diameter and 1.5 mm in height) was used as the 3D-printed model, since such annular structures comprise the basic structural unit in complex constructs such as tracheal tubes and cortical bone and also serve as a better choice to examine the 3D constructs’ stability during handling and media change. Four bioinks, namely, CA, 0.9GMPs-CA, 2.7GMPs-CA, and 4.5GMPs-CA, were used for printing hNCs. The detailed printing parameters are presented in Table S3. The printing pressure for various bioinks was quite similar to 20–21 kPa for CA and 0.9GMPs-CA and 22–23 kPa for 2.7GMPs-CA and 4.5GMPs-CA (Table S3), with all bioinks yielding physically stable constructs. The constructs were cultured in chondrogenic differentiation media for 28 days (4 weeks) and then characterized for cartilaginous tissue formation.

**Figure 7:**
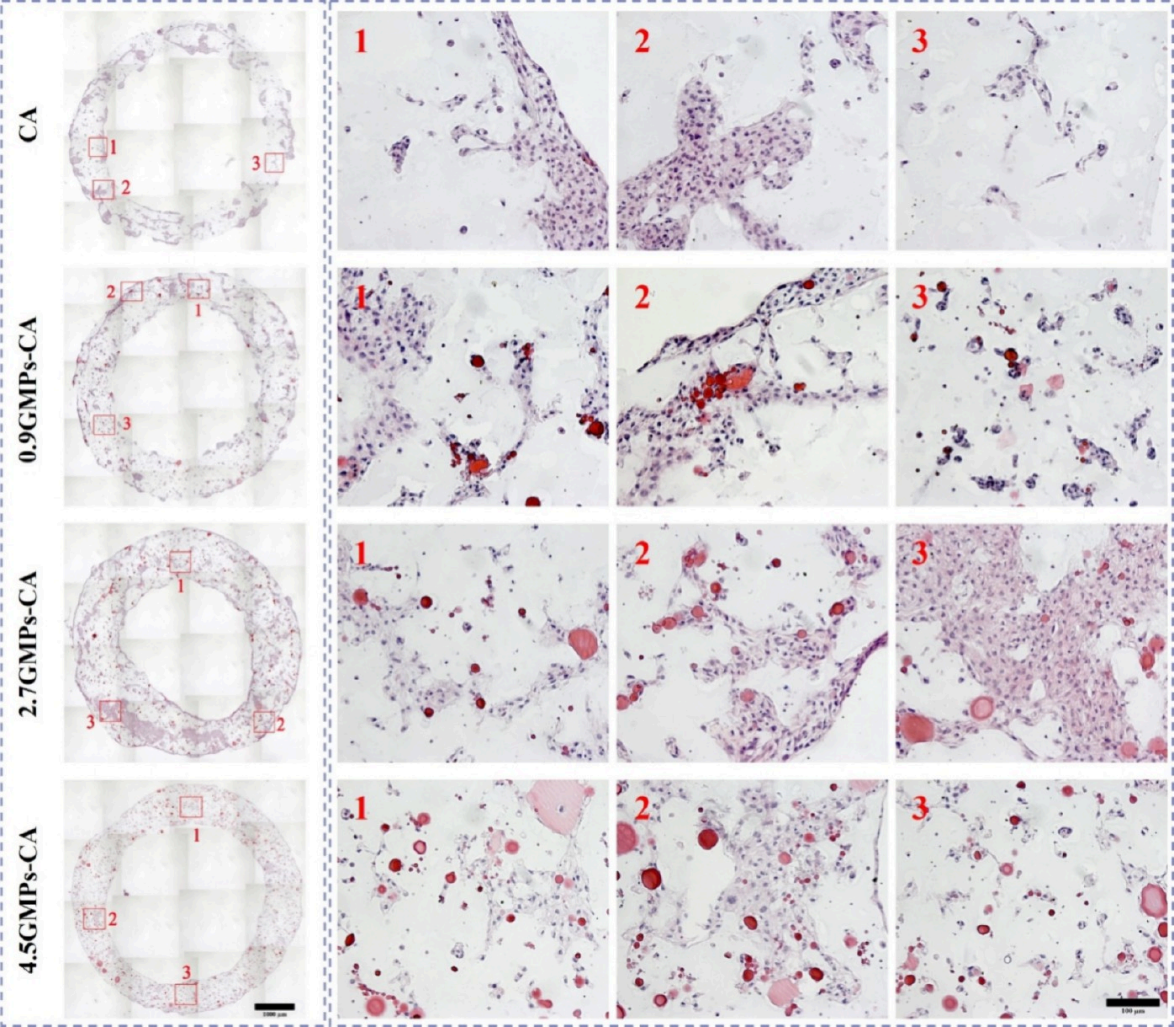
Effect of GMP volume fraction on the aggregation behavior of hNCs within GMP-CA printed constructs. Optical micrographs of H&E-stained sections of printed hNC-constructs after 28 days in vitro culture in chondrogenic differentiation medium. (Hematoxylin for nucleus (purple or dark) and eosin for ECM (pink)). The rows of figures labeled by Arabic numerals 1–3 are magnification of respective regions denoted by squares in the corresponding image in the left column. Note that cells in CA aggregated in large islands. In contrast, cells in GMPs-CA were organized in smaller clusters that distributed more uniformly throughout the constructs. Left scale bar = 1000 μm; right scale bar = 100 μm. (see supplementary Figures S5 for area analysis).

Hematoxylin and eosin (H&E) staining revealed that in CA and 0.9GMPs-CA constructs, hNCs migrated predominantly toward the construct’s periphery and formed clusters eventually. However, an increasing number of small clusters formed surrounding the GMPs in 0.9GMPs-CA. In comparison, hNCs within the constructs with higher GMPs (volume fraction) ratios (2.7- and 4.5GMPs-CA bioinks) assembled more frequently into relatively smaller, elongated populations, dwelling more homogeneously throughout the constructs, and as a result, large regions devoid of cells were infrequent. Moreover, fewer large clusters were found along the edges compared to CA and 0.9GMPs-CA groups (Figure 7), suggesting hNCs preferred to stay within the constructs rather than migrate outward. This observation was further confirmed by quantification of the area fraction occupied by cell clusters, which clearly showed the emergence of smaller clusters and the absence of large clusters with increasing GMPs volume fraction (Figure S6).

To further elucidate the spatial relationship between GMPs and newly deposited ECM, the printed 3D constructs were subjected to critical-point drying and imaged using SEM. As visualized in Figure S7, across all GMPs-CA samples, GMPs were surrounded by filamentous ECM proteins, most likely collagen fibers, which were particularly prominent in the samples with higher GMPs ratios. This outcome confirmed GMPs’ role in attracting cells and anchoring their secreted ECM. The more GMPs dispersed within the printed 3D constructs, the more centers of ECM deposition regarded as foci of tissue genesis were formed. GMPs can also serve as a source of soluble signals. We have recently shown that the release of FGF-2, a key growth factor for hNC proliferation, can be modulated over a few hours to a few days using the charge characteristics of CA from matrices of both CA/agarose and CA/gelatin,^45^ and via a biomimetic approach using negatively charged polysaccharides.^46^ Based on our previous studies where the codelivery of FGF-2 and TGB-β1 was shown to enhance chondrogenesis in periosteal cells,^47^ one can envision exploiting CA-gelatin blend microparticles for spatiotemporal delivery of FGF-2 and TGF-β1 to stimulate hNCs’ expansion and enhanced chondrogenesis.

### GMPs-CA Bioinks Contribute to Homogeneous ECM Deposition and Tougher Cartilaginous Constructs

**Figure 8:**
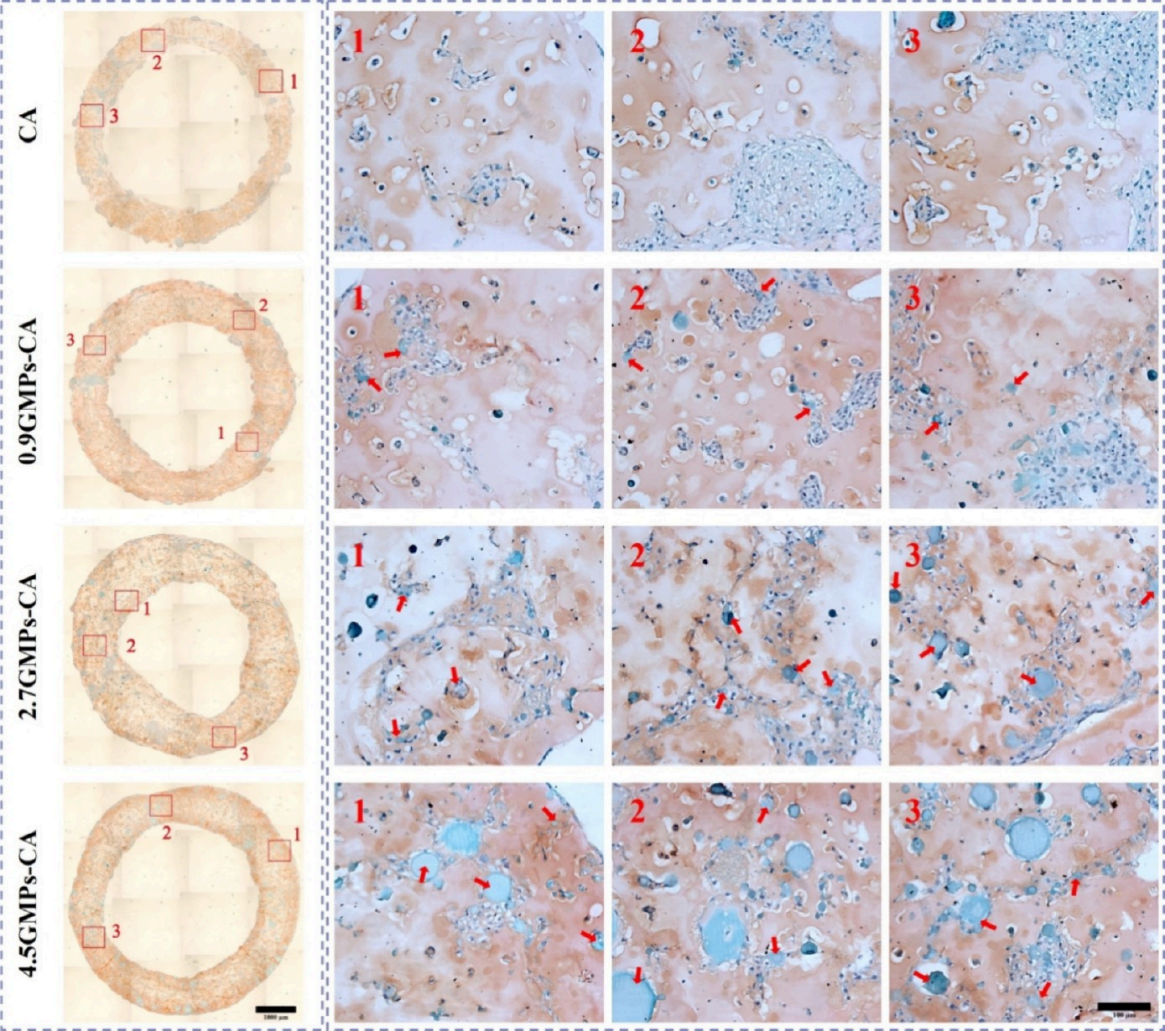
GAG deposition in printed GMPs-CA constructs. Optical micrographs of Safranin-O-fast green-stained paraffin sections of printed hNC-constructs after 28 days of in vitro culture in chondrogenic differentiation medium. Deep orange areas represent sGAGs-rich regions stained by Safranin-O, and spherical green areas represent GMPs (representative GMPs were denoted by red arrows). The rows of figures labeled by Arabic numerals 1–3 are magnification of respective regions denoted by squares in the corresponding image in the left column. Left scale bar = 1000 μm; right scale bar = 100 μm.

**Figure 9:**
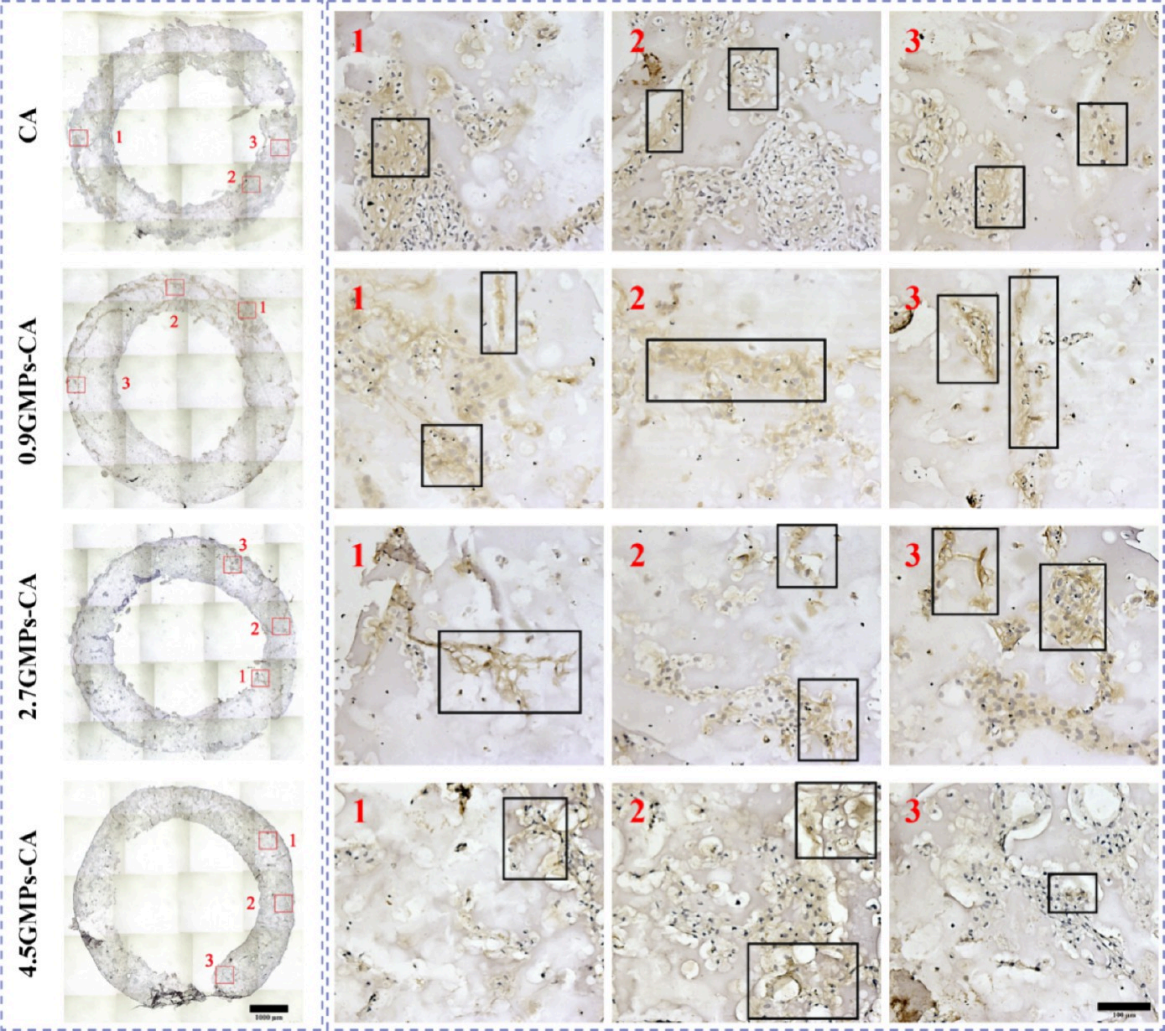
Deposition of collagen II by hNCs in GMPs-CA printed constructs after chondrogenic stimulation. Representative optical micrographs of IHC staining for collagen type II (COL2) for sections of printed hNC-constructs after 28 days culture in chondrogenic differentiation medium. The rows of figures labeled by Arabic numerals 1–3 are magnification of respective regions denoted by squares in the corresponding image in the left column. Note the strong staining in cells within black boxes, suggesting a chondrogenic phenotype. Left scale bar = 1000 μm; right scale bar = 100 μm.

Hyaline cartilage exhibits biphasic viscoelastic behavior, which arises from the presence of sulfated glycosaminoglycans (GAGs), which serve as fixed charges within the type-2 collagen matrix,^48^ thus providing the osmotic activity needed to develop hydrostatic pressure that contributes to the mechanical attributes of hyaline cartilage.^49^ Therefore, the qualitative and quantitative assessment of sGAG deposition is considered an important characterization parameter for engineered cartilage constructs.^50,51^ To ascertain the quality of the cartilaginous constructs, paraffin sections were stained using Safranin-O to reveal regions rich in sGAGs. Although the CA portions also stained light orange due to their acidity, sGAGs could be distinguished by a deeper orange color, particularly around cells or within the cell clusters (Figure 8, first row). In the CA constructs, spherical large cell clusters were prevalent, resembling the morphology observed in pellet culture.^28^ These cell clusters, or pellets, contained densely packed hNCs enveloped by sGAG-rich tissues. In spite of some small cell aggregates and isolated cells outside the hNC pellets, quite a large cross-sectional area was devoid of both cells and sGAGs. In the 0.9GMPs-CA group, the Safranin-O staining revealed similar outcomes (Figure 8, second row). Meanwhile, in the 2.7- and 4.5GMPs-CA samples, elongated and narrow cell- and sGAG-rich areas were more frequent as opposed to large spherical cell clusters, which could be attributed to the attachment and spreading of hNCs within the 3D environment. Furthermore, these sGAG-rich tissues were found either surrounding the GMPs (stained as green) or as connections among GMPs (Figure 8, third and fourth rows). In addition to sGAG, IHC staining for collagen type II (COL2), another pivotal marker for cartilage, revealed that COL2-positive regions indicated by brown staining were in accordance with the sGAG-rich regions (Figure 9).

**Figure 10:**
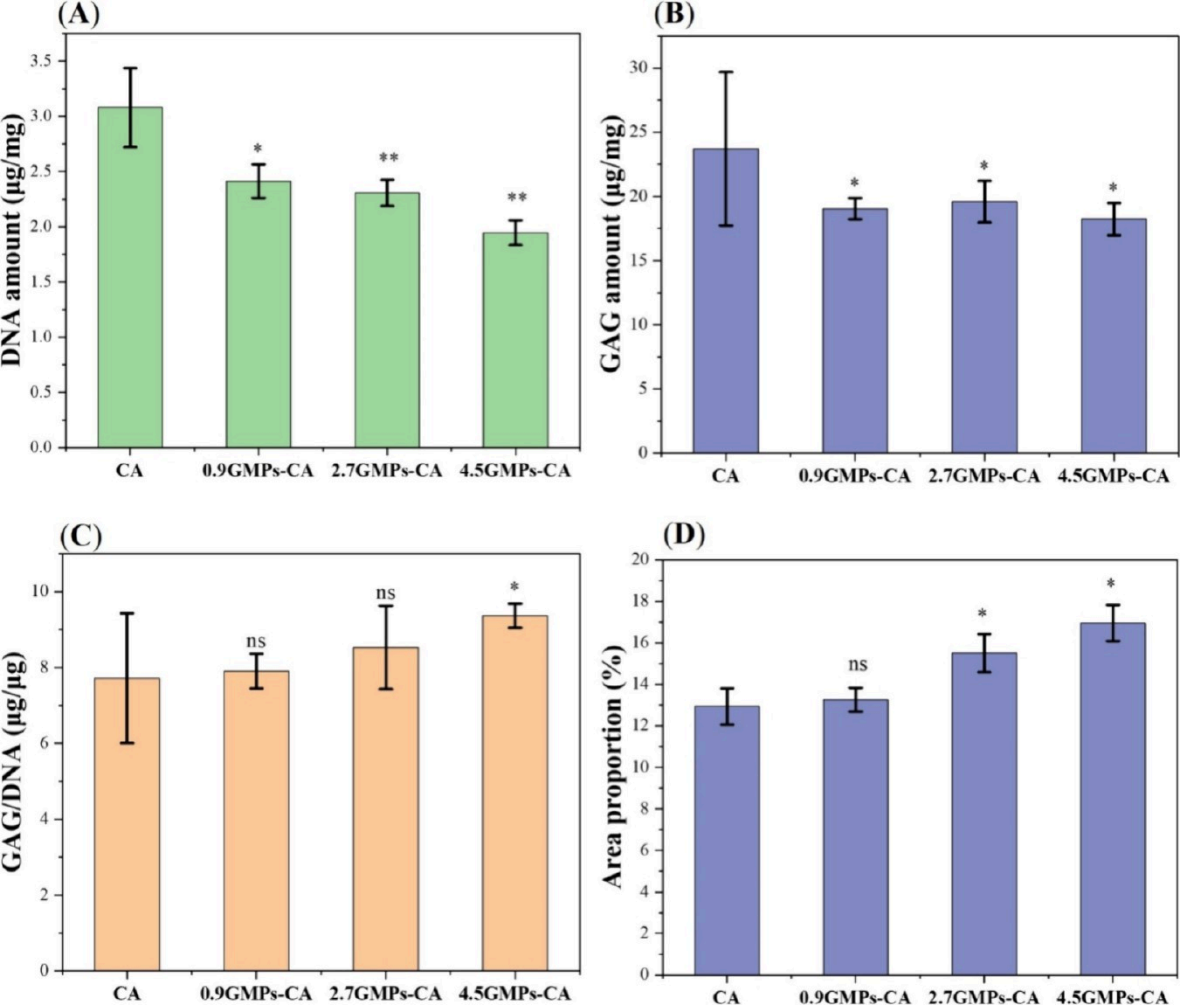
Effect of GMP volume fraction on GAG secretion and ECM production by hNCs within GMPs-CA printed constructs. (A) DNA amount, and (B) GAG amount per weight of dry construct. (C) GAG per DNA analysis of the constructs. (D) Quantification of the area of ECM based on the HE staining. (*N* = 3, ns: no statistically significant difference, *p ≤ 0.05, **p ≤ 0.01).

Quantitative analyses for GAG and DNA were conducted to further evaluate the efficiency of chondrogenesis on a cellular level within the constructs printed using different bioinks. The CA group exhibited the highest levels of DNA and GAG per dry weight of the sample, while the 4.5GMPs-CA group demonstrated the lowest levels (Figure 10A,B). This observation can be rationalized on the basis of the total solid content in CA versus 4.5GMPs-CA. Although both constructs have the same weight percent of CA at 8%, the total mass in the 4.5GMPs-CA is actually over 50% higher due to the contribution of the GMPs. Since GMPs are cross-linked, they are likely not degraded during the duration of the in vitro culture, and therefore continue to skew the dry weight in these samples to higher values. Furthermore, due to the absence of a sampling time point immediately postprinting, normalization to baseline DNA values could not be done. Thus, longitudinal sampling and analysis involving collecting samples at multiple time points will be necessary in the future to better describe how hNCs grow within GMPs-CA bioinks. Nevertheless, the amount of GAG per hNC (reflected by DNA quantity) in 4.5GMPs-CA groups was the highest, with no significant differences observed among the other three groups (Figure 10C). In addition to these analyses, we computed the proportion of the area of cartilaginous tissues relative to the entire cross-section of the constructs from the H&E staining. As the ECM-positive regions were stained pink and CA was not stained in any color, the pink-stained area could reflect the cartilaginous tissue. Based on this analysis, the 4.5GMPs-CA constructs contained over 16% cartilaginous tissue, followed by the 2.7GMPs-CA group with approximately 15%. Both of these values were significantly higher than those of the other two groups, which were each estimated at 13% (Figure 10D). While the increase in cartilaginous tissue is rather modest, its cumulative effect on tissue quality and mechanical properties might be noticeable.

**Figure 11:**
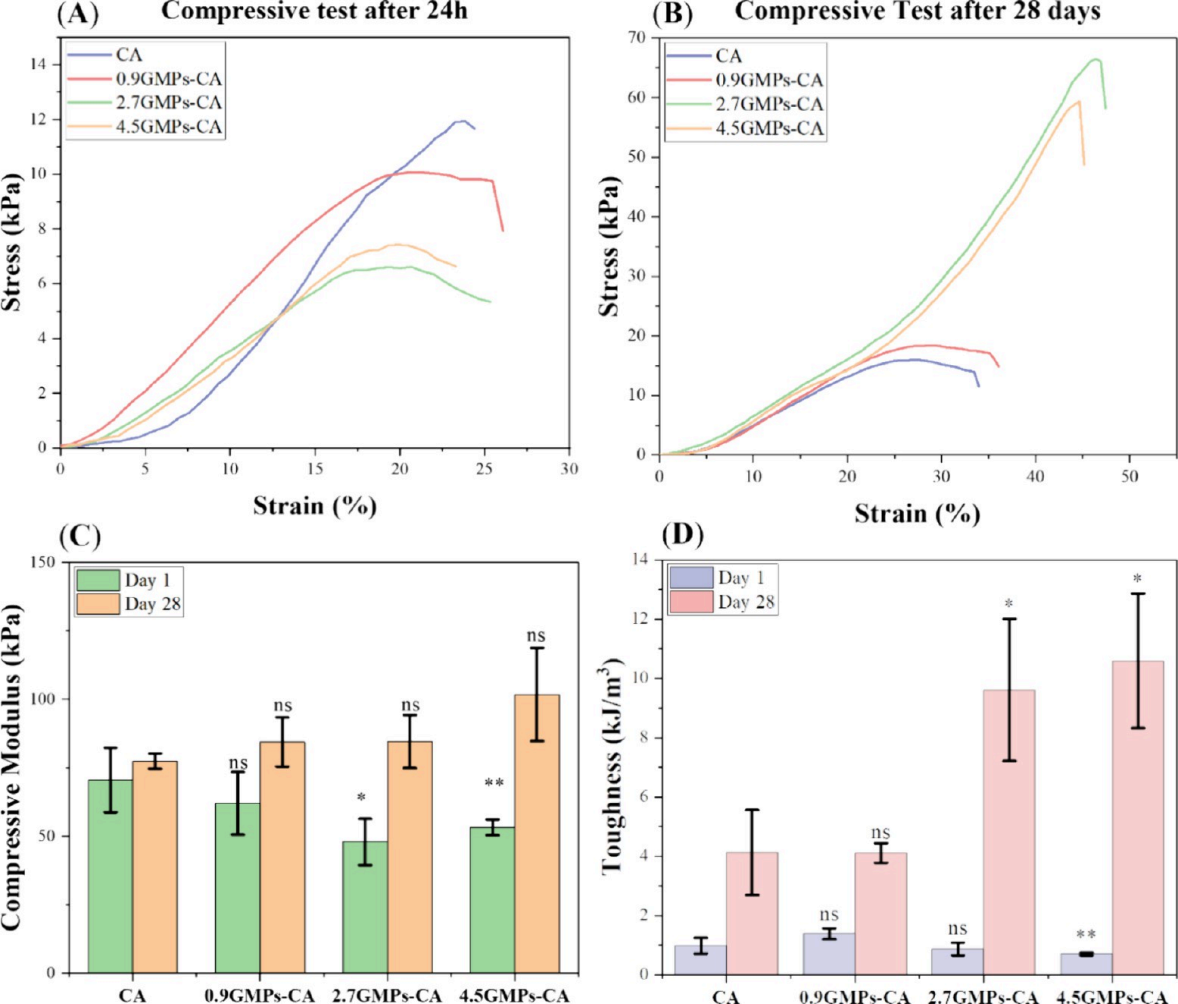
Toughness of GMPs-CA constructs after 28 days culture. (A,B) Strain–Stress curves of printed hNC-constructs harvested 24h after printing and after 28 days culture in chondrogenic differentiation. (C,D) Comparison of compressive modulus and toughness of printed constructs after 24h and 28-day culture in chondrogenic differentiation medium (*N* = 3). As a reference, the toughness of native cartilage ranges between 10 KJ/m^3^ and 800 KJ/m^3^ (ns: no statistical difference, *p ≤ 0.05, **p ≤ 0.01).

Since the physiological function of articular cartilage is to dissipate axial stress in the human body, it is essential to ascertain the mechanical properties of the constructs to evaluate how much energy they can absorb. Toward this end, the printed constructs were tested using an unconfined axial compressive test at two time points: 24h postprinting and after 28 days of chondrogenic differentiation (Figure 11A,B). Initially, at 24h, the CA and 0.9GMPs-CA constructs were stiffer than the constructs printed with 2.7- and 4.5GMPs-CA (Figure 11A,C), with the 4.5GMPs-CA constructs demonstrating the lowest energy tolerance (Figure 11D). This could be explained by the introduction of GMPs interrupting the entanglement of the CA polymers, making the high-GMPs ratio constructs softer and more fragile. After 28 days of chondrogenic differentiation, there was no significant difference in compressive moduli among all groups (Figure 11B,C). However, the toughness of 2.7- and 4.5GMPs-CA constructs was 9.61 ± 2.40 and 10.59 ± 2.27 KJ/m^3^, respectively, approaching the lower end of native cartilage’s toughness (10–800 KJ/m^3^) (Figure 11B,D). This was a significant outcome as tough constructs could more possibly withstand postimplantation stresses. These findings were also closely related to the histological outcomes. Although larger chondrogenic cellular fractions were formed in the CA and 0.9GMPs-CA constructs, these stiff focal areas could not counterbalance the soft parts devoid of ECM and dominated by CA, thus leading to heterogeneity in mechanical properties. In comparison, the long, narrow chondrogenic tissues distributed more uniformly and densely throughout the 2.7- and 4.5GMPs-CA constructs function in concert to reinforce and strengthen the entire construct. An extended in vitro culture may lead to improvements in the current outcomes, as in some studies, culturing up to 16 weeks was shown to yield better outcomes.^52^ In vivo, one can envision that extensively dispersed foci of chondrogenesis may initiate rapid in vivo remodeling, which is essential for cartilage regeneration.^53^

## Conclusions

In this study, a paradigm for promoting cell attachment and function in inert bioinks through the incorporation of proteinaceous microparticles is presented. Insoluble, bioactive GMPs were synthesized in various size distributions using a water-in-oil emulsion technique followed by glutaraldehyde cross-linking. Integration of GMPs into biologically inert carboxylated-agarose-based bioink at optimal concentrations resulted in bioinks that exhibited excellent printability, which also promoted cell attachment. From a cartilage engineering standpoint, the presence of GMPs within the printed 3D constructs was found to promote adhesion and spreading of hNCs and mitigate the formation of large hNC clusters. Furthermore, GMPs-hNC interactions promoted the establishment of chondrogenesis foci, which connected to one another to develop a more homogeneous cartilaginous tissue in vitro. The GMPs-CA bioink system holds promise as a viable biomaterial for the customized fabrication of 3D-printed cartilage engineered tissues.

## Materials and Methods

### Synthesis of Carboxylated Agarose

Carboxylated Agarose (CA) was synthesized as previously described.^24^ Briefly, 10 g of native agarose type 1 (GeneOn, Germany) was dissolved in 800 mL of deionized water (DI H_2_O) in a three-necked round-bottom flask equipped with a magnetic stir bar and pH meter at 90 °C. When the agarose was completely dissolved, cool down the reaction system to 0 °C by an ice bath to produce a slurry of agarose gel under continuous mechanical stirring. The reactor was then charged with 206 mg TEMPO (Abcr, Germany) in 100 mL DI H_2_O, 1.5 g NaBr in 50 mL DI H_2_O, and NaOCl 27 mL (15% v/v solution) under vigorous stirring. The pH of the reaction system was adjusted to 10.8 and maintained by 0.5 M NaOH throughout the reaction. The degree of carboxylation was controlled by adding predetermined volumes of NaOH solution (0.5 M) with 28 mL. At the end of the reaction, 1.5 g NaBH_4_ in 50 mL DI H_2_O was added. Meanwhile, 600 mL ethanol was added with 1 M HCl, 20 mL to maintain pH ≈ 8 last 1 h. The CA was precipitated by the sequential addition of 150 g NaCl and 2 L ethanol, and the product was collected by vacuum filtration and extracted using ethanol. Residual ethanol was removed by extensive dialysis against water, and CA was obtained as a white fibrous solid upon freeze-drying overnight.

### Preparation of CA-Gelatin Blend Hydrogel Film and Fibroblast Culture

#### Preparation of Hydrogel Films

CA was blended with Gelatin type A (GelA) (G2500, Sigma-Aldrich, Germany) in Dulbecco’s phosphate-buffered saline (DPBS) (Gibco, Germany) with a total weight/volume percentage of 12%. The ratios and concentrations of CA and gelatin are described in Table S4. The liquid CA-gelatin hydrogel was loaded into Petri dishes to form hydrogel films with 200–400 μm thickness. The cross-linking was carried out using a 0.5% glutaraldehyde solution for 3 h. After cross-linking, the hydrogels were washed with DPBS several times to remove unreacted glutaraldehyde, and the films were removed from the bottom of Petri dishes and processed into discs (Ø 6 mm) using a disposable Biopsy Punch (Integra Miltex, Germany). For cell culture, the films were transferred into a 48-well plate with an agarose-coated bottom.

#### Fibroblast Culture

NIH-3T3 fibroblasts cell line was procured from the BIOSS Toolbox (Signal Haus, University of Freiburg) and genotyped and verified to be free of mycobacteria. Fibroblasts were expanded in an expansion medium consisting of high glucose DMEM (4.5% glucose, Glutamax and Sodium pyruvate (Gibco, Germany) supplemented with 10 v/v % fetal bovine serum (FBS) (Gibco, Germany) and 1% Penicillin/Streptomycin (PAN-Biotech, Germany). Fibroblasts were detached using trypsin-EDTA and triturated to yield a suspension in the expansion medium and then seeded at a density of 50,000 cells per well and cultured in expansion medium, with media change every other day. The morphology of the cells was observed using an optical microscope (Observer A1, Zeiss, Germany).

### Preparation of Gelatin Microparticles

GMPs were prepared using the oil-in-water emulsion method as illustrated in Figure 2A and previously described.^31^ Briefly, 10 mL of gelatin solutions at (5, 10, and 15% w/v) were prepared in DPBS and added dropwise to a beaker containing 25 mL of paraffin oil and 1 mL of Span-80 (Sigma-Aldrich, Germany) at 60 °C and a stirring speed of 400 rpm. After 30 min, the emulsion was cooled rapidly in an ice bath while maintaining the stirring at 400 rpm to induce physical gelation. Then, 3 mL of 1.67% glutaraldehyde solution (Sigma-Aldrich, Germany) was added to initiate chemical cross-linking, and the solution was stirred at 400 rpm for an additional 60 min. The GMPs were dehydrated by adding 50 mL of precooled acetone and collected by vacuum filtration, repeating the acetone wash several times to remove residual oil and glutaraldehyde. The washed GMPs were vacuum-dried at room temperature overnight. Finally, the GMPs were resuspended in DPBS and 100 μL of the GMPs dispersion was pipetted into 10 mL of Coulter Isoton II diluent, and particle size distribution was measured using a Multisizer 3 (Beckman Coulter, Germany).

### Detection of Residual Glutaraldehyde

The residual glutaraldehyde concentration in CA-GelA hydrogels and GMPs was quantified using a Schiff base reaction with glycine. Briefly, CA-GelA hydrogel discs (25 mg) were cast and cross-linked with 5000 μg/mL glutaraldehyde, while GMPs (25 mg), after thorough rinse, were weighed into a well plate. Both the hydrogels and GMPs were incubated in 500 μL of DPBS per well at 37 °C. At specific time points, all the supernatant was collected, and 50 μL of which was mixed with 150 μL of 0.5 M glycine. The mixture was incubated at 60 °C with shaking at 500 rpm for 1 h to facilitate Schiff base formation. Fluorescence intensity was measured using a Biotek Synergy HT microplate reader (Biotek Instruments, USA) with excitation at 485 nm and emission detection at 520 nm. Each time after the collection of the supernatant, fresh DPBS was added to the samples. The residual glutaraldehyde concentration was calculated using a glycine-derived standard curve.

### Human Nasal Chondrocytes Isolation and Expansion

Human nasal chondrocytes (hNC) were isolated from the nasal cartilage of healthy donors. The tissue samples were obtained during orthopedic procedures under the general informed consent of the University Hospital Basel and in accordance with the local ethical committee’s regulations (University Hospital Basel, Ref Number 78/07). hNC and BFP-hNC were expanded with chondrocyte expansion medium containing 87% DMEM with high glucose 4.5%, Glutamax and Sodium pyruvate (Gibco, Germany), 10% FBS (Gibco, Germany), 1% Penicillin/Streptomycin (PAN-Biotech, Germany), 1% 1 M HEPES (PAN-Biotech, Germany), 1% MEM-NEAA (100×, PAN-Biotech, Germany) and 1 ng/mL TGF-β1 (R&D systems), 5 ng/mL FGF-2 (R&D systems). hNC and BFP-hNC were expanded in 2D and were harvested by regular trypsinization.

### Preparation of GMPs-CA Blend Bioink

The GMPs dispersion was centrifuged at 1600 rpm at 4 °C for ten minutes, and then, the supernatant was removed. The precipitate was sterilized in 70% ethanol under magnetic stirring at room temperature for 24 h. Subsequently, the dispersion in ethanol was centrifuged, and sterilized DPBS was added to the dispersed GMPs again. The schematic illustration of the preparation of GMPs-CA bioinks is shown in Figure 1B. Lyophilized CA powders were weighed into a syringe at a concentration of 16% (w/v), and DPBS was added to dissolve the mixture in a 95 °C water bath. The CA was then filtered through a 0.45 μm syringe filter (Sarstedt, Germany), reheated to remove bubbles, and kept at 45 °C for 2 min. GMPs, pipetted from dispersions, were centrifuged and added into the CA, and mixed to reach a final fraction of 8% (w/v) and 0.9%, 2.7%, or 4.5% (w/v) GMPs, then thermally equilibrated for another 5 min. The hNC and BFP-hNC cells were harvested and centrifuged at 800 rpm for 5 min, with the supernatant removed, and the pellets resuspended in 100 μL FBS per volume of bioink. The hNC and BFP-hNC suspensions were then uniformly mixed into the GMPs-CA at concentrations of 6 × 10^7^ cells/mL for hNC and 3 × 10^7^ cells/mL for BFP-hNC.

### Rheological and Mechanical Characterization of Bioinks

All rheological and mechanical tests were performed using a Kinexus-PRO+ rotary rheometer (Malvern, U.K.). The samples were prepared with noncell bioink for the rheological tests, and measurements were carried out using a 4° cone-and-plate or a parallel-plate geometry with a diameter of 20 mm. The compression tests were performed using cell-laden bioinks using a flat plate geometry, 20 mm in diameter. To simulate the bioprinting process, samples were heated in a 95 °C water bath for 1.5 min and equilibrated at a 45 °C water bath for 5 min. The samples experienced a round-trip change from 90 to 4 °C at 1.0 Hz, 0.1% shear strain, at a rate of 5 °C per minute during the temperature sweep. In time sweep, shear rate ramp, and shear stress ramp tests, the samples were measured at 33 °C. In compression tests, samples were compressed at a rate of 0.1 mm/min at room temperature.

### Bioprinting and Culturing of Printed Constructs

#### Printing Parameter Preparation

The constructs were printed using an Inkredible-2 bioprinter (Cellink, Sweden), incorporating various in-house modifications, including an aluminum temperature-control module for heating the print nozzle and a cooled print bed. The generation of G-code was performed using Slic3r (GNU Affero General Public License) with parameters provided by the bioprinter manufacturer. Preparation of GMPs-CA bioinks was carried out using a protocol described previously.^15,27^ Following thermal equilibration at 45 °C for 5 min, the bioink was transferred into a preheated cartridge equipped with a G24 nozzle set at 33 °C and allowed to rest for an additional 10 min to attain thermal and physical equilibrium before printing on the printer bed set to a temperature of 6 °C.

#### Printing of the Trigonal Pyramidal Structure

The construct was designed in Autodesk Inventor Professional (2024), exported as an.stl file, sliced within Cellink Heartware, and then printed exactly as detailed above. 2.7GMPs-CA bioink was used for printing the structure. The bioink was supplemented with 0.2 wt % activated charcoal powder (Activated charcoal, NORIT A Supra, Acros Organics) purely for enhancing the visibility of the otherwise translucent ink. In order to compensate for the reduction in total area toward the end of the print, the printing was gradually slowed down manually during the printing to 80% of the printing speed in the end to accommodate the small size of the structure.

#### 3D-Printed Constructs for In Vitro Culture

To investigate chondrogenesis in printed constructs, ring-shaped constructs (5 mm inner diameter and a 6 mm outer diameter height of 1.5 mm) containing hNC at a density of 6 × 10^7^ cells/mL were printed. For imaging cell bioink interactions and cell behavior, square sheets (5 mm × 5 mm and 0.5 mm in height) were printed, and hNCs constitutively expressing BFP (BFP-hNCs) were incorporated at a density of 3 × 10^7^ cells/mL. The sheets were imaged using an ECHO fluorescence microscope (ECHO, San Diego, USA).

#### Culture of Printed Structures

The printed structures were transferred into a 48-well plate with agarose-coated bottom, and 500 μL chondrogenic differentiation medium was added and the constructs were incubated at 37 °C, 5% CO_2_ with media change every 2 days. The chondrogenic differentiation medium contained 95% DMEM, 1% HEPES, 1% MEM NEAA, 1% penicillin/streptomycin, 1% ITS-IV (100×, PAN-Biotech, Germany), 1% HSA (100×, University Hospital Freiburg, Germany) with 10 ng/mL TGF-β 1, 10^–7^ M dexamethasone (Sigma, Germany) and 0.1 mM Ascorbic Acid (Sigma, Germany).

### Morphological Characterization of GMPs and GMPs-CA Printed Structures

GMPs were dehydrated with acetone, dried in a vacuum oven, and then sputter-coated with gold and images using a Quanta 250 FEG (U.K.) field emission scanning electron microscope (SEM) at an accelerating voltage of 20 kV and a chamber pressure of 100 Pa. Likewise, printed GMPs-CA structures with and without cells (after 28 days of chondrogenic culturing) were fixed overnight in 3.7% formaldehyde at room temperature. Samples were then dehydrated at room temperature using an ethanol series (2 h per ethanol series). To ensure preservation of ultrastructure, dehydrated samples were dried using a supercritical carbon dioxide (CO_2_) critical point dryer (EM CPD300, Leica, Germany) using an automated program provided in the instrument. The samples were freeze-fractured in liquid nitrogen and coated with gold using a high vacuum sputter coater and then imaged using SEM.

### GAG and DNA Quantification

After 28 days of culture, printed constructs were lyophilized and their dry weight was measured. Each sample was digested in 500 μL 125 μg/mL papain cocktail solution (Sigma P3125, Germany) for 16 h at 60 °C. After that, the sample was centrifuged at 10,000 rpm for 10 min, and the supernatant was used for GAG and DNA analysis.

#### GAG Analysis

The amount of GAG was quantified using a dimethylmethylene blue (DMMB) method. Briefly, 100 μL of supernatant was transferred into 1 mL of DMMB solution and placed on a shaker for 30 min at room temperature. Subsequently, samples were centrifuged at 12,000 rpm for 10 min to sediment the GAG as a purple pellet. The supernatant was removed, and 700 μL of decomplexion solution was added to each pellet, and the samples were heated at 60 °C for 20 min. The reacted mixture was then pipetted into a 96-well plate (200 μL/well) and the absorbance was measured at 656 nm using a Biotek Synergy-HT plate reader (Biotek Instruments, USA) microplate reader. The GAG amount was calculated from a standard curve established using a known GAG amount.

#### DNA Quantification

5 μL of the digested supernatant was diluted with 95 μL 1× TE buffer. 1× TE buffer was prepared by dilution of 20× TE buffer in RNA/DNA-free water (Promega, USA). Then 100 μL of the diluted sample solution was added to 100 μL PicoGreen working solution (working concentration 5 μL/mL PicoGreen (200×, Molecular Probes, USA)) in 1× TE buffer. Plates were incubated for 5 min in the dark, and the fluorescence was measured using the program for DNA quantification in the plate reader using an excitation wavelength of 485 nm and emission at 528 nm. The DNA amount was calculated from the standard curve established using a known amount of DNA.

### Histological Characterization

Printed constructs were fixed, dehydrated with gradient alcohols, and embedded in paraffin, and paraffin-embedded blocks were cut into 5 μm thick sections using a microtome (Microm HM360, Zeiss, Germany). For staining, sections were first deparaffinized using xylene and rehydrated using gradient alcohols. For hematoxylin and eosin (H&E) staining sections were treated with Mayer’s hematoxylin (direct for use, AppliChem, Germany) and 0.25% Eosin Y (Sigma-Aldrich, Germany) solution. For GAG staining, sections were stained with Fast Green solution (AppliChem, Germany) and 0.1% Safranin-O. For type II collagen immunohistochemistry, sections were first subjected to enzymatic antigen retrieval by hyaluronidase and Pronase, followed by staining with primary antibody (rabbit anti collagen type II, GTX20300, GeneTex, USA) and secondary antibody (Biotinylated goat anti rabbit IgG antibody, ab6720, Abcam, USA) after blocking. A DAB detection kit (Vector Laboratories, USA) was used to reveal regions of positivity. Afterward, the sections were counterstained with hematoxylin and dehydrated, cleared, and mounted with permanent mounting media and then coverslipped.

### Quantification of the Area Fraction of ECM

Slides stained with H&E were utilized for quantification of the ECM area fraction. Initially, the H&E-stained slides were digitized and imported into ImageJ software (National Institutes of Health, USA), where they were converted into 8-bit grayscale images. Subsequently, the region of interest within these images was delineated by applying an appropriate threshold to highlight the area. The contour of the printed construct was then traced using the lasso tool. Finally, the ECM area fraction was determined by calculating the ratio of the area of interest to the total area of the sectional construct.

### Software

The schematic illustrations were drawn using Adobe Illustrator, and the images were analyzed using Fiji ImageJ. Graphs were generated using Origin graphing software, and all figures we composed in Microsoft PowerPoint.

### Statistical Analysis

Statistical analysis was carried out using a statistical package embedded in Origin graphing software, and statistical significance was determined using Tukey’s multiple comparison test. A *p*-value of ≥0.05 was considered statistically insignificant (ns), and *p*-values ≤ 0.05 were considered statistically significant (*p ≤ 0.05, **p ≤ 0.01).

## Supporting Information Available

The Supporting Information contains the following Figures S1–S7, and Tables S1–S4. Behavior of NIH-3T3 fibroblasts CA and GelA gels; NIH-3T3 fibroblasts adhesion on cross-linking CA- gelatin hydrogels; concentration of residual glutaraldehyde detected from supernatant of CA-GelA hydrogel; relationship between theoretical mass and actual mass of GMPs obtained from GMPs suspension; temperature sweep of CA bioink containing GMPs-09 and GMPs-34 dispersions at different concentrations; distribution and characterization of ECM islands within the printed constructs; microscopic characterization of GMPs-CA printed constructs containing hNCs; theoretical mass of GMPs in a given volume of GMPs suspension; storage modulus of GMPs-CA-bioinks during temperature sweep at various temperatures during the print process; parameters for 3D bioprinting; and Ratios of CA and GelA component in blend hydrogels (PDF)
