# Supplementary material for: Incorporation of Cross-Linked Gelatin Microparticles To Enhance Cell Attachment and Chondrogenesis in Carboxylated Agarose Bioinks for Cartilage Engineering
Source: ACS Appl Mater Interfaces. 2025 Apr 7;17(15):22293–307. doi: 10.1021/acsami.5c00077 (PMC12012782; doi:10.1021/acsami.5c00077)
Supplement: Supplementary file 1 — am5c00077_si_001.pdf [file am5c00077_si_001.pdf]

Supporting Information

Incorporation of crosslinked gelatin microparticles to enhance cell attachment and chondrogenesis in carboxylated agarose bioinks for cartilage engineering

Yi Qian[1], Yawei Gu[1*], Fabian Tribukait-Riemenschneider[1], Ivan Martin[2], V. Prasad Shastri[1,3*]

1. Institute for Macromolecular Chemistry, University of Freiburg, 79104 Freiburg, Germany;
2. Tissue Engineering Laboratory, Department of Biomedicine, University Hospital Basel, University of Basel, 4031 Basel, Switzerland;
3. BIOSS – Centre for Biological Signalling Studies, University of Freiburg, 79104 Freiburg, Germany

*Corresponding authors: Prof. Prasad Shastri, prasad.shastri@gmail.com

Dr. Yawei Gu, gu_yawei@163.com

Keywords: gelatin microparticles, carboxylated agarose, bioink, cartilage engineering, cellular niches, 3D bioprinting

Supplementary Figures

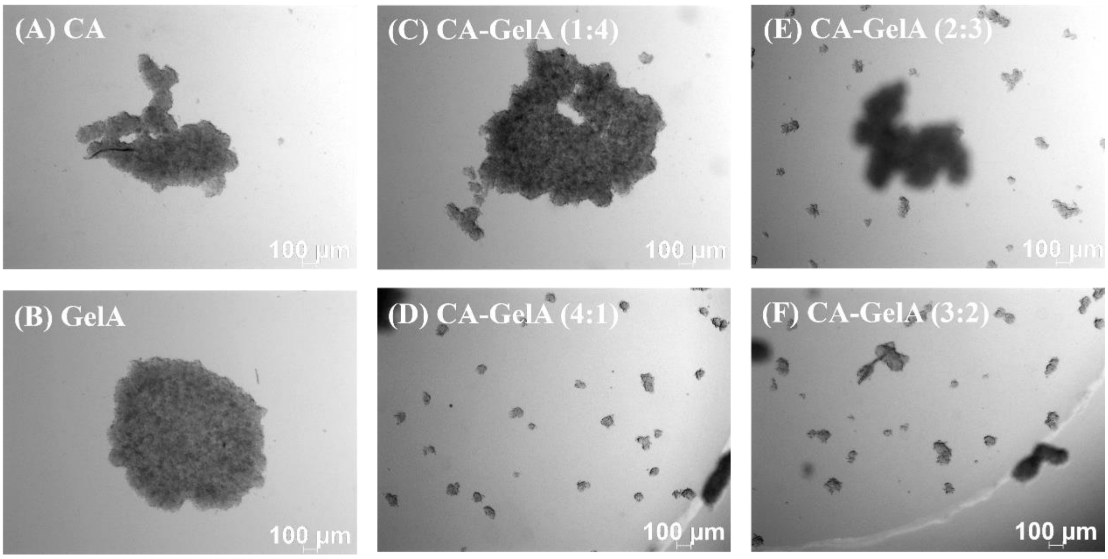

Figure S1. Behavior of NIH-3T3 fibroblasts 24h after seeding on hydrogels of **(A)** CA, **(B)** GelA, and **(C-F)** and mixture of with various ratios CA and GelA.

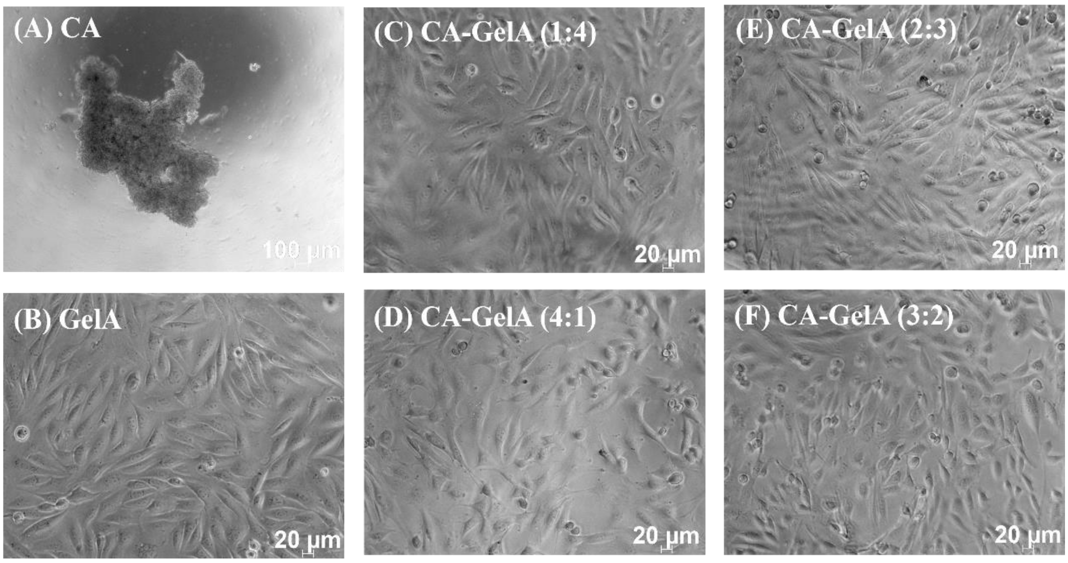

Figure S2. Behavior of NIH-3T3 fibroblasts on **(A)** CA, **(B)** GelA crosslinked with glutaraldehyde, and **(C-F)** CA-GelA hydrogels of various ratios crosslinked with glutaraldehyde. Stabilization of gelatin within CA-GelA hydrogels provides cell adhesion sites on the hydrogel surface, promoting the adhesion and spreading of NIH-3T3 fibroblasts. The images were capture 48 hours after cell seeding.

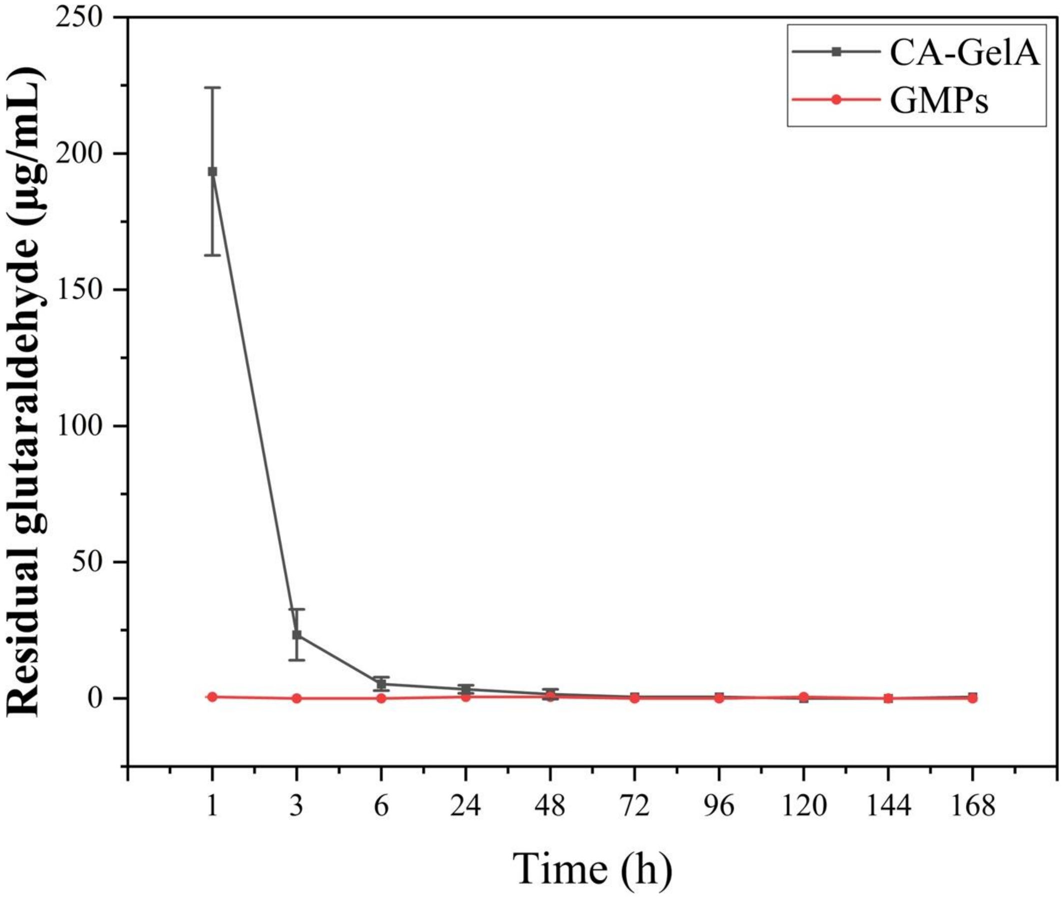

Figure S3. The concentration of residual glutaraldehyde detected from supernatant of CA-GelA hydrogel crosslinked by glutaraldehyde or GMPs over the incubation period. (N=3)

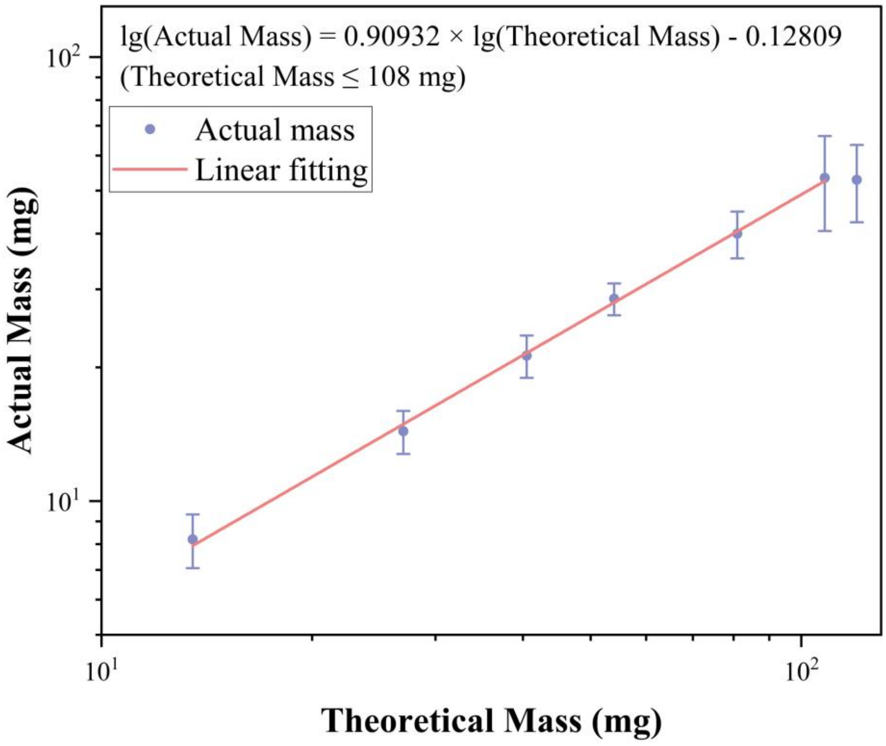

Figure S4. Relationship between theoretical mass and actual mass of GMPs obtained from GMPs suspension. The red line represents a linear regression fit and the equation of this fit is shown on the top of the graph. (N=3)

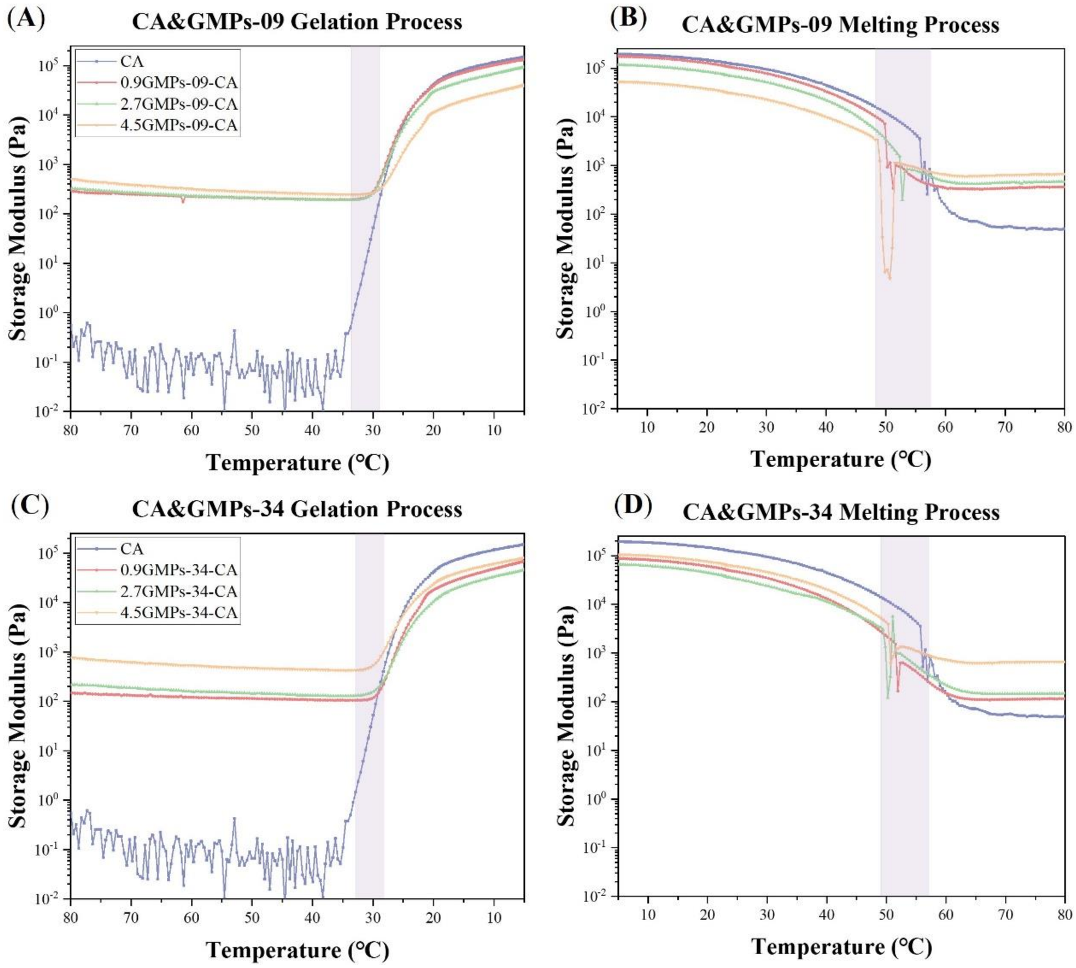

Figure S5. Temperature sweep of CA bioink containing GMPs-09 and GMPs-34 dispersions at different concentrations (w/v%), showing (A, C) sol-gel and (B, D) gel-sol transition of CA and GMPs-CA bioinks. The large fluctuations seen in the CA storage modulus curve before sol-gel transition in A and C is due to the onset of dynamic physical crosslinks.

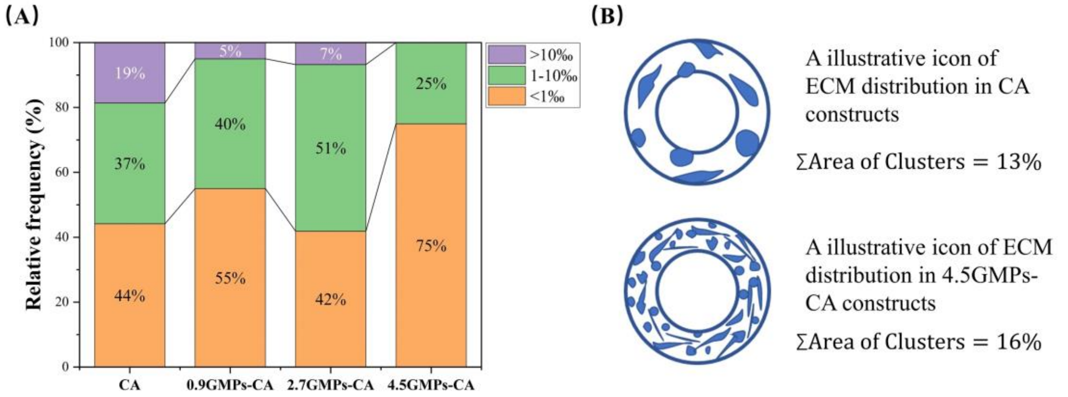

Figure S6. The distribution of ECM islands within the printed constructs, characterized by specific area percentages, indicates distinct modes of ECM formation. (A) A stacked 100% bar chart shows the accumulation of individual ECM islands falling within defined area range (n=3). The orange, green and light purple bars correspond to single ECM islands occupying less than 1‰, between 1-10‰, and greater than 10‰ of the entire construct area, respectively. (B) Illustrative models that demonstrate the ECM islands across both CA and 4.5GMPs-CA constructs.

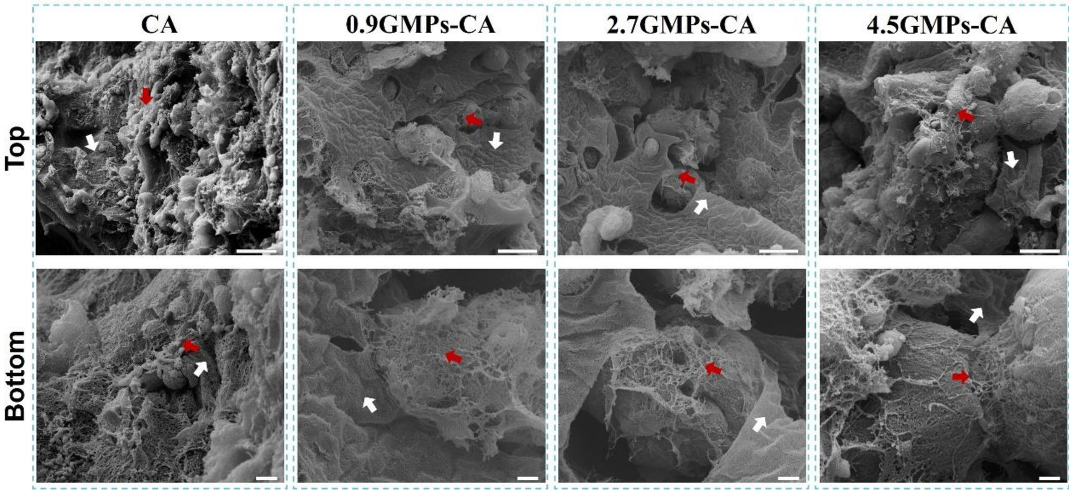

Figure S7. Microscopic characterization of GMPs-CA printed constructs containing hNCs revealing cell attachment niches. SEM micrographs of critical-point dried CA and GMPs-CA printed constructs after 28 days in vitro chondrogenic differentiation. The adherence of cells to GMPs surface is identified by red arrows denoting the ECM deposited by the attached hNCs. The CA component is identified by the white arrows. Scale bar: 15 μm for top row and 2 μm for bottom row.

Supplementary Tables:

Table S1. Theoretical mass of GMPs with corresponding volume of GMPs suspension at a concentration of 75mg/mL and measured actual mass of GMPs. (N=3)

Theoretical mass of GMPs (mg)	Volume pipetted from GMPs suspension (μL)	Actual mass of GMPs (mg) (N =3)
13.5	180	8.20 ± 1.14
27	360	14.37 ± 1.59
40.5	540	21.27 ± 2.32
54	720	28.57 ± 2.35
81	1080	40.03 ± 4.82
108	1440	53.47 ± 12.90
120	1600	52.93 ± 10.49

Note: *GMPs were dispersed in DPBS to form GMPs suspension.*

Table S2. Storage Modulus of GMPs-CA-bioinks during temperature sweep at various temperatures during the print process. Syringe: 45 °C, Print head 33 °C, Print bed: 6 °C. (N=3)

	**Name**	**45°C (Pa)**	**33°C (Pa)**	**6°C (Pa)**
	**CA**	0.57 ± 0.74	1.29 ± 0.43	(1.23 ± 0.25) × 10^5
	**Wt-% GMPs**	**(Pa × 10^2)**	**(Pa × 10^2)**	**(Pa × 10^5)**
**GMPS-09-CA**	0.9	1.24 ± 0.84	1.23 ± 0.83	0.97 ± 0.28
	2.7	1.93 ± 0.68	1.89 ± 0.61	0.79 ± 0.09
	4.5	2.96 ± 0.38	2.84 ± 0.42	0.55 ± 0.15
**GMPS-22-CA**	0.9	1.03 ± 0.56	1.12 ± 0.61	0.95 ± 0.18
	2.7	2.67 ± 1.94	3.10 ± 2.02	0.86 ± 0.16
	4.5	3.40 ± 1.41	3.84 ± 1.50	1.06 ± 0.14
**GMPS-34-CA**	0.9	0.58 ± 0.47	0.55 ± 0.46	0.63 ± 0.07
	2.7	0.84 ± 0.47	0.85 ± 0.39	0.56 ± 0.14
	4.5	2.01 ± 2.24	1.97 ± 2.07	0.76 ± 0.02

Table S3. Parameters for 3D bioprinting

Group name	GMPs swelling mass (mg/mL)	GMPs swelling volume (μL/mL)	Cell concentration (Million/mL)	Cell volume (μL/mL)	Printing pressure (kPa)
CA	\	\	59.28	235.83	20 ~ 21
0.9GMPs-CA	77.42	45.83	65.83	245.83	20 ~ 21
2.7GMPs-CA	231.50	141.67	61.83	233.33	22 ~ 23
4.5GMPs-CA	367.75	219.17	60.32	227.50	22 ~ 23

Note: *GMPs swelling mass and volume means the mass of GMPs after reswelling and supernatant removal account in Bioink.*

Table S4. Ratios of CA and GelA component in blend hydrogels.

Group name	CA w/v (%) in DPBS	Gelatin w/v (%) in DPBS	CA: GelA
GelA	0%	12%	0: 1
CA-GelA (1: 4)	2.4%	9.6%	1: 4
CA-GelA (2: 3)	4.8%	7.2%	2: 3
CA-GelA (3: 2)	7.2%	4.8%	3: 2
CA-GelA (4: 1)	9.6%	2.4%	4: 1
CA	12%	0%	1: 0

Note: *GelA refers gelatin type A.*
